# Adopting and embedding home sensors in social care: findings from a mixed methods, rapid evaluation

**DOI:** 10.3310/nihropenres.14011.1

**Published:** 2025-08-22

**Authors:** Joseph Wherton, Stephanie Stockwell, Nikki Newhouse, Stuart Redding, Anna Louise Todsen, Caroline Potter, Stavros Petrou, Sonja Marjanovic, Sara Shaw

**Affiliations:** 1 University of Oxford, Nuffield Department of Primary Care Health Sciences, Oxford, England, UK; 2 RAND Europe, Cambridge, England, UK

**Keywords:** Technology-enabled care; remote monitoring; social care; evaluation; rapid evaluation

## Abstract

**Background:**

The growing pressure faced by adult social care in England has fuelled interest in technology-enabled care (TEC). This includes the use of sensor-based technology to monitor activity patterns for ‘proactive’ interventions and care. However, evidence on its effectiveness and use is limited to feasibility pilots, as opposed to business-as-usual. Working with three local authorities using home sensors, we sought to define good practices and draw transferable lessons on implementing and embedding this technology in routine care practice.

**Methods:**

Across all sites, we interviewed 51 staff and system stakeholders, 19 service users, and family/informal caregivers. We also used secondary data to determine the feasibility of a full economic study. The analysis was guided by the NASSS (non-adoption, abandonment, and challenges to scale-up, spread and sustainability) framework to explore factors influencing implementation and sustained adoption of the technology in use.

**Results:**

Home sensors were used across multiple care contexts (assessment, reablement, and long-term care monitoring). Perceived value and impact included an increase in service user independence and safety, family/informal caregiver reassurance, identifying healthcare needs, providing more holistic and objective assessments, and supporting dialogue regarding care needs. However, evidence of the impact across these areas was limited, and we were unable to obtain the data required to undertake an economic analysis. Key issues to consider for sustained adoption include the materiality and dependability of the technology, compatibility with service users and their care networks, workforce knowledge and confidence, inter-organizational routines and coordination work, and strategic alignment.

**Conclusion:**

Our findings indicate the need to acknowledge the labor-intensive process of embedding and adapting the use of home sensors for proactive care. Decision makers need to focus on how to support and resource incremental and system wide-changes, with particular attention paid to ensuring technology dependability, ‘wrap around’ support, workforce knowledge and skills, co-adaptation of inter-organization routines, cross-stakeholder collaboration, and evaluation capabilities.

## Plain English summary

The growing pressure faced by adult social care in England has fuelled interest in using technology to help people remain independent and safe at home. This includes technologies that monitor people’s activities within the home, identify patterns and changes to provide reassurance to family/informal carers, and inform care decisions.

While these technologies have the potential to support safety, independence, and early intervention in health and social care, there is little evidence of their advantages and disadvantages. There is also a lack of guidance on how to introduce and use such technology in care services.

We worked with three local government councils in England, where such technology is being used in social care. We interviewed the staff and other key individuals involved in providing the technology. We also interviewed service users and family/informal caregivers about their experiences of using the technology. We also sought to collect information on how it affected the overall cost of care provision.

The interviewees highlighted many ways in which the technology helped monitor and care for people living at home. However, it was difficult to assess whether this provided good value for service users and social care in terms of the resources invested versus the benefits achieved. We identified the key factors to consider when using such technology in social care. This included setting up and maintaining the technology, fitting it within people’s lives, helping care staff develop skills and confidence using the technology, developing clear processes for provision, and forming good relationships across the different organizations involved.

In summary, using technology can help improve service user well-being; however, technology alone cannot achieve this. It must be used within social care services where people (care professionals and family/informal carers) can deliver appropriate support and engage with the insights that the technology can provide.

## Introduction

Adult social care provides a wide range of services designed to assist people who are older and people living with disabilities or physical or mental illness to maintain independence and well-being. These services include personal care (e.g., support with washing, dressing, eating) and helping people stay active and social within their communities. They can be provided within people’s homes, day centers, and residential care. Support can be short-term, with the aim of maximizing independence and eliminating the need for ongoing support, or longer term and provided on an ongoing basis.

Extensive funding cuts to the public sector and continued social care workforce shortages, coupled with increased demand from an aging population, pose significant challenges in delivering adult social care services. Funding cuts have also impacted unpaid carers (who contribute care equivalent to 4 million paid care workers and without whom the social care system would collapse), as fewer received paid support (27% in 2021/22 compared to 31% in 2015/16), although more received signposting, advice, and information (56% in 2021/22 compared to 50% in 2015/16) ^1^. The growing pressures faced by adult social care in England have led people to seek innovative solutions to meet the scale and nature of demand for social care, including technology-supported solutions. The use of digital technology and technology-enabled care (TEC) in adult social care is perceived by many to not only help the system operate more efficiently, but also to improve the quality of care and outcomes for those requiring care, and to support people to live independently in their own homes for longer ^2– 5^.

Telecare (continuous remote monitoring of people at home using indoor sensors and personal trigger alarms) has been widely embedded within social care provision for many years as a means of shifting the location of care away from residential care services to people’s own homes ^4^. Over the last decade, there has been a proliferation of digital devices (and new providers) that offer the potential to support *proactive* care, that is, the use of aggregated data of activity patterns to intervene early and ahead of the crisis point ^3, 6^. A proactive model is intended to reduce the occurrence of exacerbations that could lead to crisis events. With the increasing breadth of digital solutions alongside the analog-to-digital switchover across UK telecoms by 2025, there has been growing interest from commissioners and services to see how technology, including home sensors and analytics, can be used for personalized and cost-effective care ^7^.

The use of home sensors for proactive care can be broadly categorized into three groups: i) for *assessment review,* used ad-hoc and on a case-by-case basis to provide insight into individual needs to inform care package assessments and improve independence, whilst taking into account solutions already in place in that person’s life; ii) for *reablement,* a short-term use of monitoring (e.g. 6-weeks) following an event, such as hospital discharge post-surgery, to monitor progress and personalize care packages, whilst supporting independence, and iii) for *long-term monitoring,* used longer term to monitor people and detect changes in behavior that may require an urgent response (e.g., lack of movement) or provide insight into gradually changing conditions (e.g., changes in eating, sleep, toilet behaviors) ^6^.

However, the care sector is still at a low level of maturity in the application and adoption of such technologies and the development of proactive care models. While there is a lot of interest in proactive telecare, it has not become mainstream in social care. This rapid evaluation sought to advance our understanding of the role of home sensors in supporting proactive approaches to care, with a particular focus on how they can be effectively implemented (the efforts put in to help the uptake), sustained (continued use routinely over time), and scaled-up (supporting widespread adoption) ^8, 9^. In the following section, we summarize the current literature on the evidence base for such technology within England’s social care sector before setting out the overarching aim and evaluation questions.

### Learnings and gaps in the current literature on the use of home sensors for proactive care in England

For our targeted scoping review of existing literature, we focused on the deployment and evaluation of ‘connected care’ platforms (i.e. data from a range of non-intrusive sensors around the home are aggregated and analyzed for changes in service users’ day-to-day living patterns) within English social care services. This review of both academic and grey literature builds on our previous horizon scanning and stakeholder engagement to outline the current national terrain on the use of telecare to support *proactive* models of social care delivery. Further details on the scoping review method and findings can be found on the project website ( www.phc.ox.ac.uk/research/decide).

Overall, we found that the evidence-base on the implementation and evaluation of home sensors for proactive care was extremely limited. The five academic articles we identified described three evaluations, each of which considered evidence from multiple stakeholders and settings ^3, 10– 13^. A general review of proactive telecare services using smart home technology in the UK ^10, 11^ drew on primary interviews with stakeholders (mainly the technology designers/suppliers and social care housing providers) and a limited number of interviews with older people. An evaluation of Phase 2 of the Technology for our Aging Population: Panel for Innovation (TAPPI) project described how six testbed sites had implemented a range of care technologies, including three sites that had piloted proactive remote monitoring ^12^. A recent rapid evaluation of proactive remote monitoring within adult social care was conducted by the Birmingham, RAND, and Cambridge Evaluation (BRACE) Centre, which included two local authorities and a national charity provider of social care services as case sites ^3, 13^.

The 16 grey literature articles identified reflected a variety of stakeholder perspectives, including sector leaders in adult social care ^14– 17^, local government ^18^, and technology-enabled care ^16, 19^. Initiatives to implement proactive remote monitoring within specific local government settings were identified ^20– 25^, with some evaluation evidence available for Hertfordshire ^24^ and Kent ^20^. Within the context of integrated care systems, implementation efforts were also identified in a home care agency ^22^ and in collaboration with NHS partners ^23^. One technology supplier has produced or sponsored multiple reports that highlight a range of case studies and emerging issues within the sector ^17, 26– 28^, which are highly informative but also at greater risk of bias for a favorable review of proactive remote monitoring technologies and the supplier’s specific products.

Across the reviewed articles, there were few examples of robust evidence from independent evaluation. Many of the grey literature reports provided some information on outputs, outcomes, and/or projected impacts, but they did not contain clear evidence in support of the claims (i.e., the methods that supported the findings were not described). The academic literature provides some evaluation evidence through more robustly described methods, but most data are in the form of qualitative stakeholder interviews, with more representation from suppliers and providers of the technologies rather than service users.

All of the reported case studies were for the initial implementation stages, with a small number of service users (usually 30 or less). We found no examples where proactive remote monitoring had already been scaled up to the level of a standard service offer within local government organizations, and only one reference to scale-up in progress ^15^. Most evidence of evaluation was smaller-scale and qualitative rather than capturing system-wide quantitative data.

The design and analysis of our rapid evaluation informed by three key insights from the literature. First, there was a consensus on how proactive telecare within adult social care is framed to address system-level challenges. Policy makers, strategic decision makers within social care organizations, and technology suppliers were in alignment with the potential outcomes that these technologies could support: discharging patients from hospital as quickly as possible without compromising on safety, tailoring care packages to real-time needs in order to maximize efficiency in the context of chronic social care workforce pressures, and avoiding or delaying escalation to more intensive forms of care (e.g., moving into residential care) through earlier intervention. However, there remained tension in prioritizing system-level outcomes within an ethos of personalized care. If service users and the people in their care networks do not benefit from using the technology at a personal level, then uptake could remain low and aspirational system-level outcomes would not be realized.

Second, there was a mismatch between the articulation of system-level goals and current approaches for evaluating preventative telecare service models. The demonstration of system-level efficiencies, cost savings, and maintained/improved quality of life for target user groups requires large-scale data that capture a range of health and care outcomes over time. However, we found no examples of quantitative data on the long-term outcomes used to evaluate these technologies. Independent evaluation was limited to the few available academic sources and a small number of grey literature reports, providing mostly qualitative data on strategic decisions and early experiences of trialling technology. Most evidence of the impact was presented within the grey literature as individual case examples and unverified projections of cost savings. To effectively evaluate proactive remote monitoring technologies within social care, a wider range of data must be captured.

Third, the literature most strongly reflects the perspectives of sector leaders, organizational decision-makers, and technology suppliers rather than end users. It provided some emerging evidence of benefits to service users and their families, notably a sense of reassurance when additional care is not needed but confidence that carers will be alerted quickly if the situation changes and needs emerge. However, the evidence on end user experience of the technology remains sparse, with some indication of organizational difficulties in sustaining use. The technology prompts new roles, responsibilities, and (often hidden) workflows for a range of end users, particularly care staff, who are expected to engage with the data produced by remote monitoring systems. The values and experiences of all end users – the social care service users, their family carers, front-line care professionals, and any other staff responsible for data monitoring and response – must therefore be understood.

In summary, the shift to preventative telecare models involving proactive remote monitoring technologies has gained considerable momentum since the Covid-19 pandemic. The value proposition of the technology’s potential for addressing priority system-level challenges has been driven strongly by technology suppliers and strategic decision-makers, in line with overarching policy aims. There is emerging evidence of implementation outcomes from numerous pilot studies, but currently no evidence of local authorities in England (in their role as statutory providers of adult social care) that have demonstrated the scale-up and sustained use of these technologies beyond the pilot. There is a notable gap in the evidence of on-the-ground experiences of using these technologies, particularly regarding how different stakeholders respond to the information presented on data dashboards for each system. There was also a lack of available quantitative data to support the robust evaluation of longer-term, system-level outcomes and the impacts of implementing preventative telecare models. These gaps informed the mixed-methods design for collecting and analyzing new primary evidence within the three case study sites selected for this rapid evaluation.

### Aims, objectives and evaluation questions

Against this background, we sought to study the use of home sensors for proactive care within adult social care services in England, in order to define good practices in the implementation and use of such technology, and draw transferable lessons that can inform sustained adoption at scale. The evaluation was guided by the following questions.

1) What constitutes in-home sensing in the context of supporting social care, who is it for, and how does it help provide care to service users?
2) What impact and value does this have across the care system and how could this be locally evaluated and monitored in the future?
3) What does sustained adoption at scale look like within the context of technology-enabled in-home monitoring within social care and how can it be achieved?
4) What structures and resources (financial, organizational, technical, and human) are needed to achieve this goal?

## Methods

### Origins of study and evaluation approach

The evaluation was conducted as part of the Digitally Enabled Care in Diverse Environments (DECIDE) program, a partnership between the University of Oxford and RAND Europe, undertaking rapid evaluation of technology-enabled remote monitoring across health and social care ^29^. The topic of focus was identified and refined through initial scoping meetings with national stakeholders and guidance from the DECIDE steering committee (a cross-sector group bringing perspectives from across the four UK nations). This study was informed by the tradition of developmental evaluation involving an emergent approach that captures data that can inform ongoing developments and focus ^30^. Working in partnership with three organisational case sites in England (local authorities and collaborating organisations using/supporting in-home monitoring within social care), we explored the multiple system-wide influences on implementing and embedding home sensors for proactive care within routine practice.

### Case sites

We initially engaged with six local authorities where in-home sensing was being implemented and used as part of service transformation. From these, we selected three local authorities as collaborating case sites to ensure maximum variation with regard to the technology platforms and approaches to proactive monitoring, geographical contexts, and populations served (with inequalities being a key theme of interest). Data collection sources, protocols, and the associated governance requirements were established for each site.

The evaluation was conducted across three case sites (pseudonyms for the sites and technologies were used to maintain anonymity). All three sites use one or more technology platforms consisting of a range of in-home sensors (e.g., motion, doors, fridge, bed/mat, smart plugs), an activity dashboard accessible via computing or mobile devices, and machine learning capabilities and/or customizable parameters to detect changes in usual routines and predefined activity/event notifications. Table 1 summarizes the case sites.

**Table 1.** Overview of the three case sites included in the evaluation.

Site (pseudonym)	Typical service user cohort for home sensors (n)	Technology being used	Organisation and staffing	Service set up and duration
**RIVERBOURNE** **COUNTY COUNCIL**	Aged 65+, cognitive impairment, live alone (n=100 active)	Pulse (predominant system used); Responda Monitoring & urgent alerts	Service delivery partner monitors insights and urgent alerts, covers installation, handover & maintenance	Set up in 2020; initial focus on post-hospital discharge frailty
**EASTVALE COUNTY COUNCIL**	Aged 65+, cognitive impairment; learning disabilities; those with increased risk of readmittance (n= >600 active)	KinLink Monitoring & urgent alerts	KinLink ‘wraparound’ support: installation, handover & maintenance	Set up in 2019
**STEELGATE** **CITY COUNCIL**	Aged 65+, cognitive impairment, live alone (long term monitoring only) (n=60 combined target)	Responda (long term monitoring); SafeNest (short term monitoring) Monitoring only	Responda offered via existing domiciliary care provider (monitored by care provider & council commissioning team); SafeNest offered on hospital discharge (monitored by family & SafeNest team); all installation & removal conducted by council TEC partner	Set up in 2024 as a 6-month pilot

The Riverbourne County Council has multiple districts and boroughs with a mix of relatively affluent towns and rural villages, 73% of which took part in a pilot service that started in 2021, with the service subsequently made available across the whole county. The county council’s adult social care team estimates over 700 use cases of home sensor services in the county during 2021–2024, although there are typically around 100 service users at one time. They use two different technology platforms with similar functionality: Pulse and Responda (though predominately using the former). Both of these platforms are used across different pathways and are monitored by a ‘proactive monitoring team’ alongside the traditional alarm receiving center, who reviews activity data and contacts family/informal networks regarding ‘actionable’ insights and ‘urgent’ alerts, based on changes in activity.

The Eastvale County Council is a predominantly rural county with market towns, rural areas, and pockets of high deprivation. The use of home sensors forms part of a wider TEC service, which was established in 2019, with home sensors introduced into the service model in 2020. More than 600 home sensor devices are used at any time. The technology provider, KinLink, provides all other TEC as well as home sensors, and conducts installation and monitoring. KinLink monitors urgent alerts (e.g., falls); however, continuous proactive monitoring is undertaken by the family/informal care network.

The Steelgate City Council is an urban area with a diverse population and high levels of deprivation in some inner-city areas. Home sensing has been introduced as part of a wider digital care transformation program. At the time of the study, they piloted two different technology platforms for two distinct pathways. The Responda system was used for long-term monitoring, while SafeNest was used for short-term reablement following hospital discharge.

### Qualitative data collection and analysis

The data were collected between August 2024 and April 2025. Table 2 provides an overview of the qualitative data (supplementary case summaries are included in the data repository).

**Table 2.** Overview of qualitative data and stakeholder engagement.

Data source	Data collected	Contribution to findings
**Staff interviews**	→ Interviews with 51 staff and system stakeholders, including decision-makers, commissioners, TEC service managers and practitioners, frontline care practitioners (e.g. social workers, occupational therapist), installers, coordinators, service managers, care workers, alarm response call operators. → Riverbourne = 17 → Eastvale = 17 → Steelgate = 17	→ Understanding the organisational context and use of home sensors → Understanding different roles and experiences of provision and use across the system → Understanding different perspectives and impact on work practices
**Staff system mapping**	→ Three workshops (one at each site) with 13 staff and system stakeholders across sites (TEC team managers and practitioners, frontline practitioners, decision makers, commissioners, installers, technology providers, service delivery team members). → Riverbourne = 4 → Eastvale = 4 → Steelgate = 5	→ Overview of the service delivery processes, organisational roles, systems → Gain further detail on sensor data flows and associated technology/infrastructure → Identify key resource and funding sources across the system
**Service user and family/informal carer interviews**	→ Interviews with 3 service users and 16 family/informal carers → Riverbourne = 13 (10 family/informal carers, 3 service users) → Eastvale = 3 (family/informal carers) → Steelgate = 3 (family/informal carers)	→ Understand experiences of living with the technology → Understand experiences of service delivery → Identify key challenges and areas for improvement
**Stakeholder workshop and follow-up interviews**	→ Online workshop with 25 participants (national and regional decision-makers, commissioners, TEC service managers, care providers, technology providers, PPIE representatives) → Cross-site workshop with 4 representatives from across the three sites (TEC team leads and commissioners) → Interviews with 6 national/regional stakeholders (e.g. national-level advisors, decision makers)	→ Refinement of emerging findings, particularly relating to scale up and spread and policy implications → Further insights to guide and refine analysis → Explore key issues/idea raised through stakeholder workshop, steering group and project advisory group

A total of 51 interviews were conducted with social care staff and system stakeholders (professionals from other related organizations, including technology providers, service delivery partners, and domiciliary care services). Participants were recruited through combined purposive and snowball sampling, asking the main contacts at each site and successive interviewees to connect with potential participants. This provided a diverse sample of staff and stakeholders with clinical/care and technical, managerial, strategic, and administrative roles in the delivery and use of home sensors. The staff interviews were semi-structured, conducted online, and lasted up to one hour, covering their role and use of home sensors within their organizations, implementation and delivery, perceived impact, and the value of the technology and service model.

In addition, we conducted system mapping workshops with 4–5 interviewed staff members at each case site. Workshops allowed us to develop and refine visual maps of the service user journey (to explore key steps in the delivery and use of home sensors), data flows (to explore how sensor data are shared and used across different systems and organizations), and funding (to explore different sources of funding for the service). To this end, we invited representatives with different roles and from different organizational partners (e.g., social care TEC strategy, management and administration, installation and technical support, and frontline care practice).

Service users and caregivers were identified by the staff at each case site. All were provided (or were caring for someone) using home sensors. In total, we conducted 16 family/informal caregiver interviews and three service user interviews. Most interviews were conducted with family/informal caregivers due to the service users’ lack of capacity to consent to the interview. Nearly all interviews were conducted over the telephone and lasted up to 60 minutes (two interviews were conducted at the person’s home). Participants were identified and initially approached by care practitioners on the basis that the service users were provided with home sensors.

All interviews were written using RAP (Rapid Assessment Procedure) sheets, along with selected audio transcripts where needed, using a guiding template to cover content related to the technology, use of the home sensor data, organizational processes and structures, perceived impact and experience with the technology, and challenges and enablers to implementation and use. The templates were used adaptively, depending on the background and role of the participants.

Data from staff and carer/user interviews at each site were synthesized using a structured narrative document. Narrative documents were used to support cross-case comparisons to develop a final theorization of the sustained adoption of home sensors at scale.

Emerging findings were explored and tested through stakeholder workshops with 25 attendees from across policy, health and social care, TEC and social care advisory bodies, the technology industry, third sector, and PPIE representatives. Participants were identified and invited with the support of our policy partners, project advisory, and PPIE groups. Informal interviews were also conducted with six national stakeholders: three were unable to attend the workshop and three followed up on points raised during the workshop discussion. In addition, a cross-site learning workshop with 1–2 representatives from each case site was conducted to discuss emerging findings and facilitate shared learning.

**Figure 1.**
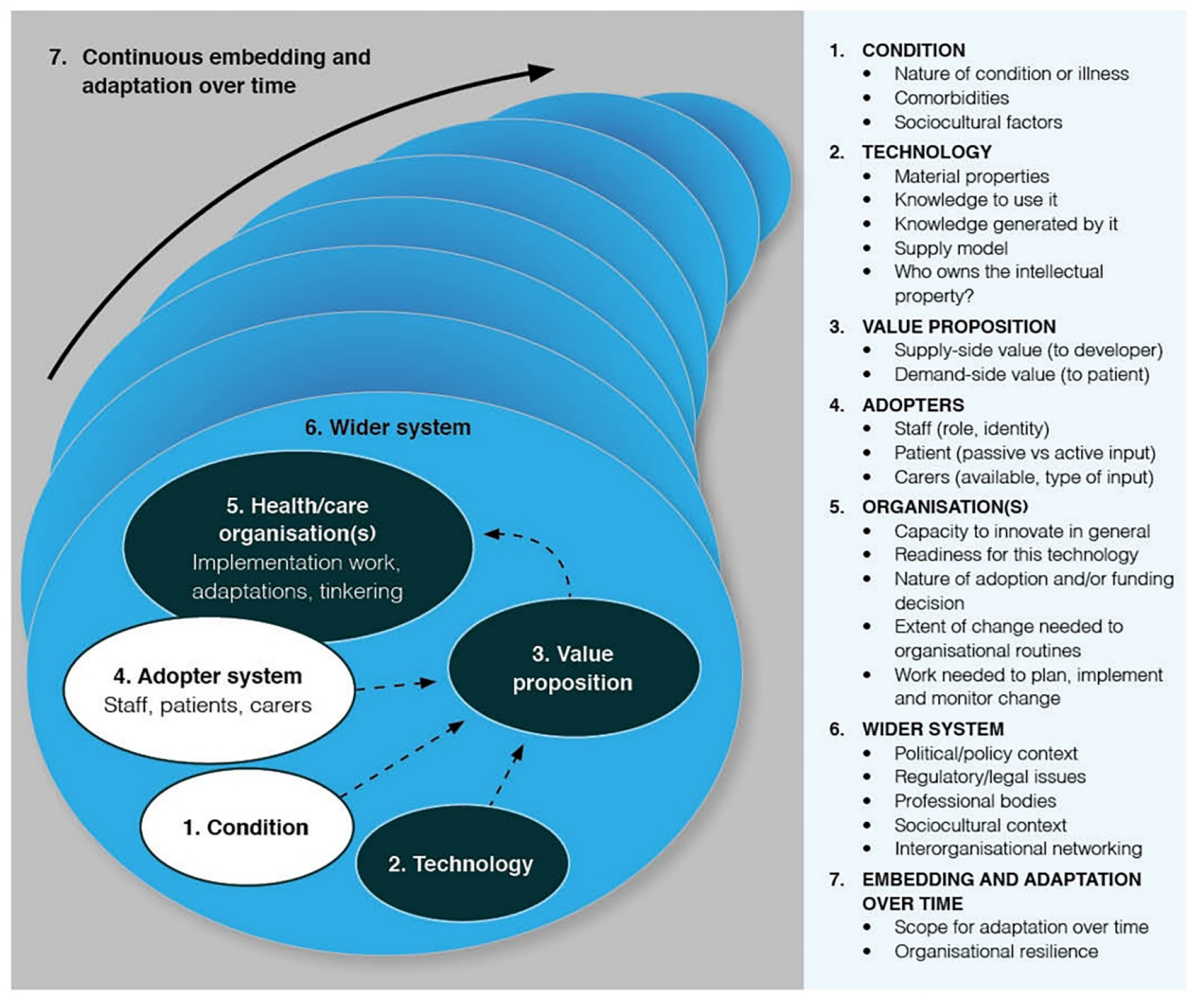
NASSS framework ^31^.

The analysis was guided by the NASSS (non-adoption, abandonment, and challenges to scale-up spread and sustainability) framework ^31^. The NASSS framework was used adaptively as an analytical tool to surface and explain the challenges and complexities in technology-supported service changes. It includes seven interacting domains: the condition or illness, technology, value proposition, adopter system (intended users), organization(s), wider system (especially regulatory, legal, and policy issues), and emergence over time (see Figure 1 below). Alongside the NASSS framework as the overarching analytical framework, we applied three main theoretical lenses to further explore the influences on the sustained adoption of home sensors in routine care practice. First, we drew on diffusion of innovation theory ^32, 33^ to further explore the organizational conditions and the work of introducing, embedding, and scaling up technology. Second, we drew on the notion of organizational routines (defined as recognizable, repetitive patterns of interdependent action carried out by multiple actors) as a source for stability and change ^34^, with a particular focus on how day-to-day work and coordination is achieved across organizational boundaries. Thirdly, we applied the theoretical conceptualization ‘information infrastructure’ (defined as the backgrounded things that other things ‘run on’) ^35^, in order to gain further insight into the invisible and taken-for-granted aspects of infrastructure (which includes technology and IT systems, but also material, social and regulatory aspects) that shape and constrain the pace and trajectory of innovation and service development.

### Economic component

We initially planned to undertake a full cost-consequence analysis. However, this was not possible because of the unavailability of the required data, coupled with the rapid evaluation timeframe. However, an analysis was conducted to explore the conditions under which a fully powered economic evaluation can be conducted in the context of proactive telecare. We sought to assess and inform approaches for monitoring implementation costs, service use, and economic and service user outcomes across sites. To this end, we worked with collaborating case-site teams to assess local data quality and availability as part of a feasibility analysis.

Drawing on the initial scoping work and emerging findings from the qualitative case studies, we sought to identify measures related to: (i) p *rovision and uptake of the technology* (demographics and variability across different population groups and with different care needs); (ii) s *ervice user outcomes* (e.g., clinical, social, and quality of life outcomes associated with the implementation of proactive telecare); and (iii) *service use and economic data* (e.g., health and social care utilization, including rates of hospitalization, A&E attendances, inpatient days, outpatient hospital visits, prescription costs, and social care package cost).

We struggled to obtain high-quality data covering all the areas we wanted to consider. For example, quality of life data were not collected by care providers. Additionally, key data were held outside of social care systems and control, for example, by healthcare providers, Occupational Health settings, and technology providers that run the services. We were unable to obtain data from these sources.

There is no unique model for the management of social care provision, nor do social care organizations use the same data systems. Therefore, it was difficult to navigate the system data specifically for our evaluation question on the economic impact of home sensors. We also faced challenges regarding information governance for data-sharing. These included uncertainties around consent, confidentiality, and legal definitions required to facilitate data sharing for the purposes of service evaluation. Our case site partners worked collaboratively to address such challenges but also faced resource limitations. In addition, when analyzing the direct impact of specific interventions, establishing a comparator and including a robust-sized sample is important, but proved difficult in our study. All of these factors impacted the quality of data we were able to access, and the timeliness of access. Despite these challenges, we were able to analyze some service users’ demographic and social care provision data from all three case sites, which came from social care management systems. This enabled us to undertake a feasibility analysis to establish what would be needed for future economic analyses.

Eastvale provided the richest set of data, which included 6399 service users – 491 sensor customers and 5908 service users who did not have sensors, dating back to 2022. The data included rich demographic data, information on the health conditions for which service users received social care support, information about the care packages service users received, dates of major events (such as time they entered the care system, changes in care packages, and dates at which they stopped receiving care), and coded reasons for any changes in care. The site had access to some data on health outcomes, but was unable to share it with us because the information was controlled by the service users’ integrated care board (ICB). The costs of specific sensor devices and their maintenance and monitoring in users’ homes were incomplete. These data were provided by technology providers, but they did not maintain full historic records for all devices allocated to users. This meant that we were unsure about the full suite of items provided to any given individual. This dataset allowed us to perform a descriptive analysis but did not facilitate cost/benefit analysis.

Riverbourne provided data on 226 users with devices, and a matched sample of 226 users who did not have home sensors. They were unable to provide data on their health outcomes or health conditions.

Steelgate provided a large dataset of 6325 customers. Of these, 1233 had a TEC device and 5092 had never used any technology but it was not possible to separate home sensor users from those who were provided with passive devices such as fall sensors (coded broadly as ‘sensors’). Additionally, as with the two other sites, it was not possible to obtain data regarding the health outcomes of service users, but they were able to provide data on the number of hospital admissions for each service user.

None of the sites routinely collected information related to the quality of life of service users. Steelgate performed a trial of home sensors with a small group of participants (up to 30); therefore, we were able to conduct a survey using a questionnaire to collect primary data. This was designed to capture demographic data, quality of life measured using the EQ-5D and the Adult Social Care Outcomes Toolkit (ASCOT), health status, and interactions with health/social care providers so that we could fully cost their care and identify any savings that could accrue as a result of their use of home sensors.

We received 18 responses to the baseline questionnaire and were able to follow up with 12 participants who responded to a second questionnaire administered approximately 12 weeks later. Sections regarding interactions with healthcare and social care providers were not completed by the majority of users, and the quality of life questions did not provide consistent results, so we were unable to draw any meaningful conclusions from this component of the study. However, our work with the staff at Steelgate produced a questionnaire that could be used in subsequent studies, and we learned about the resource requirements for a fuller study.

### Ethics and governance

The Research Governance Ethics and Assurance (RGEA) team at the University of Oxford (sponsor) classified the project as a service evaluation, and therefore did not require research ethics approval.

All participants provided informed consent. All participants were provided with an information sheet and consent form detailing the study aims, design, contacts, and data privacy. The information sheet also clarified that participants had the right to withdraw from the study at any point, without needing to give a reason. All interviews were audio-recorded and written using summary notes, with key quotes included. To protect identities, we used pseudonyms for sites and the technologies they used.

### Patient and public engagement and involvement

A dedicated project PPIE advisory group with six members was established, meeting periodically for three meetings during the project period of 12 months. Four of the six members of the group included individuals with prior experience of living with and caring for someone with TEC. The other two members were representatives from Carers UK. The group advised on study design, data collection, and analysis, as well as guiding focus on issues of equity of access and ethical considerations. Members of the group also joined the cross-stakeholder workshop and advised on written outputs and plans for dissemination.

## Findings

### Origins and use of home sensors in participating case sites

The use of home sensors within the three sites aligned with the established classifications of proactive telecare pathways, as described above, including assessment, reablement, hospital discharge, and long-term monitoring. However, there was wide variation across sites in terms of how pathways were operationalized and the extent to which home sensors were implemented and used within them.

Box 1 summarizes the diverse origins, evolution, and adaptations of the use of home sensors in the case sites.

**Box 1. Origins, evolution and adaptation in use of home sensors over time across the three participating case sites**

**RIVERBORNE COUNTY COUNCIL:** Following a report commissioned by the county council in 2020 to explore the potential for using technology in social care provision, a home sensor service was designed and piloted. The county council adult social care team had a strong relationship with a local service delivery provider who had already offered other TEC across districts and boroughs, and they collaborated to design a home sensor service model pathway, with advice from a national advisory body. This included the establishment of a ‘proactive monitoring team’ within the existing service delivery partner’s alarm response center, working separately but alongside traditional telecare call operators. The pathway initially focused on reablement of post-hospital discharge for people with frailty. However, challenges related to receiving timely information from hospitals and scheduling installations compromised the ability to install sensors quickly enough to capture baseline data, which was vital for a 6-week reablement monitoring period. After six months, the service pivoted to include more use in assessments of support needs and long-term monitoring. Once the pathway was designed, an event was held with potential technology providers, and Pulse was selected, in part due to their willingness to be agile and work collaboratively with the county adult social care team and service delivery partner. As the pilot evolved, the reporting outputs from Pulse were thought to be not as useful as anticipated by the service delivery partners, so they investigated other technology providers for reporting they desired and purchased Responda to test. Both technologies continued to be used in the service, although they predominantly used Pulse. The service evolved and adapted over time in several ways, extending beyond the initial target use case and how technologies were used. This included spreading the service beyond the pilot areas to make it a county-wide offer and developing the technology (e.g., updating dashboards to make them more user-friendly, integrating other sensors, and integrating AI capabilities).

**EASTVALE COUNTY COUNCIL:** The introduction of home sensors stems from a broader strategy to transform TEC services in response to the national shift from analog to digital infrastructure by 2025. A Digital Care Lead was appointed within the council in 2019, tasked with developing a strategy and business case centered on digital and consumer technologies. The technology provider (KinLink) was contracted to support the service, initially using touchscreen tablets to connect users with formal and informal caregivers. This coincided with the COVID-19 pandemic in 2020–2021, which accelerated adoption and unlocked further funding due to lockdown-related needs. The use of home sensors for proactive care evolved from these initial TEC initiatives during the pandemic. This focused on community referrals, in which activity data were monitored by family/informal networks and caseworkers. After the pandemic, the council procured a three-year contract with KinLink in 2022 to develop the service model, extending the catalogue of TEC and sensor devices to be integrated within the KinLink platform. This was supported by KinLink’s subcontract with a service improvement consultancy to develop a workforce engagement and training program with community practitioners. Council funding was allocated to increasing the TEC team with Digital Lead Practitioners to facilitate community referrals for assessment and long-term care. In 2023, additional internal council funding was secured for a four-month pilot of a hospital discharge pathway, specifically to recruit a Digital Lead Practitioner to be based within one of the hospitals to facilitate referrals from the wards. In conjunction, KinLink agreed to reserve a dedicated installer in order to achieve rapid deployment (24 h for TEC, and 72 h for home sensors), which was a requirement set by NHS collaborators in the interests of patient safety. Following positive outcomes from this initial pilot, the regional ICB (Integrated Care Board) agreed to continue funding two hospital-based Digital Lead Practitioners, and KinLink decided to expand a team of dedicated hospital discharge installers for this specific pathway. The team continued to grow, including the creation of a Digital Lead Practitioner post in the dementia care pathway to identify potential service users through memory services. Additionally, KinLink continued to expand its business model, taking on call operator roles, and exploring the potential for a proactive monitoring component.

**STEELGATE CITY COUNCIL:** Following an independent audit of the Steelgate City Council’s current technology offering conducted in 2024, commissioning leads took the decision to develop a new TEC service delivery model and aligned commissioning strategy. The new model intended to take a strengths-based approach (focusing on an individual's abilities, knowledge, and capacities, rather than their limitations or deficits) to optimize community care services, early discharge from hospital, and prevention of non-elective admissions, all underpinned by technology. The introduction of home sensor technologies in the form of ‘Tests of Change’ was one of nine key activities undertaken in response to the findings of the audit, guided by the overarching objective of taking a ‘tech first’ approach to optimizing adult social care services. In parallel, the Steelgate City Council intended to develop a business case for working at a council level with a strategic partner, who it perceived would provide oversight of all adult social care TEC commissioning and delivery in terms of technological solutions and expertise. Following a tender process, two home sensor providers were selected to pilot the new service for nine months: SafeNest to deliver a short-term reablement service to 30 participants recruited directly at the point of hospital discharge and Responda to deliver a longer-term service to 30 participants already in receipt of domiciliary care and recruited by one of four (invited) care providers. Both services were based on an ‘actionable insight’ model because their urgent alert capacity was not interoperable with Steelgate’s existing emergency response platform. Participants in the home sensor pilot were offered Steelgate’s emergency response ‘kit’ at no cost for the duration of the pilot but were not obliged to take up this offer.

Details of the origin and development of home sensing across case sites illuminate the convoluted work of introducing the use of home sensors within social care settings and developing associated roles and processes. The approach and focus for service delivery were shaped by both new and legacy systems, cross-sectoral partnerships, varied contracting models, and incremental additions funded through a combination of local and national sources. Social care systems are known to be diverse, fragmented, and path-dependent, shaped by historic strategic decisions that set organizations on a particular path. Consequently, the technology could not simply be ‘installed’; it had to build on, and interface with the existing infrastructure or ‘installed base’ ^35^. This led to gaps in planned and actual use and shifts in focus across care pathways, depending on technical, regulatory, social, financial, and logistical contingencies.

Across all three sites, home sensors were predominantly used for older adults with mild cognitive impairment (e.g., dementia), frailty, acquired brain injury, or learning disabilities. Decisions for use were based on the need to gain insight into levels of independence and safety that would be difficult to establish via a one-off assessment (e.g., memory, communication difficulties). This tendency was also reflected in the descriptive quantitative data captured as part of the economic evaluation component. For example, in Eastvale, 77% of service users with home sensors were aged 75 years or over and 36% were identified as having dementia. Similarly, in Riverbourne, 81% were aged 75 years or older, with over half (47%) identified as having dementia or mild cognitive impairment. It should be noted, however, that while the quantitative data may be indicative of trends, affirming what staff have told us, caution should be taken in interpreting the figures due to methodological challenges, as described in the methods section.

All service users lived at home as opposed to residential care. However, the living arrangements of service users varied depending on the affordances, functions, and configurability of the technology. In Riverbourne, most deployments were for people living alone, because the platforms could not differentiate individual activities in shared settings. In contrast, the SafeNest system in Steelgate was designed to analyze activity patterns in multi-occupancy households, constructing a picture of typical activity patterns across the home as a whole rather than per person. In Eastvale the sensors could, in some cases, be configured for shared settings using targeted sensors like bed or ‘usual chair’ mats to infer individual activity, as well as targeted night-time (bedroom) activity in shared supported accommodation for mental health and learning disabilities.

**Figure 2.**
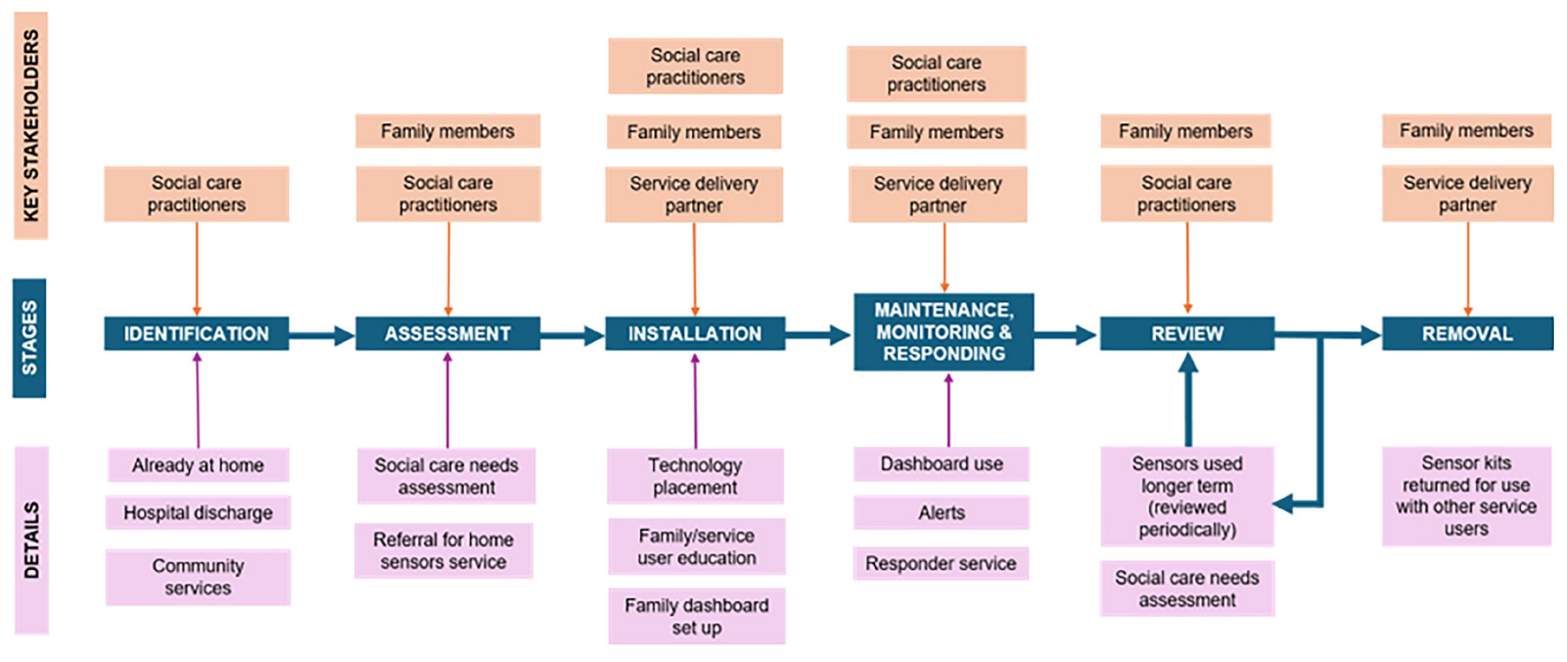
Overview of service delivery process based on system mapping across case sites.

Integrating the use of home sensors into existing workflows required significant and distributed effort across all sites, and the technology operated within a nexus of interconnected and interdependent practices across organizational boundaries and systems. Although roles and workflows varied within and across sites, system mapping revealed consistent stages and considerations in the service delivery process summarized in Figure 2, including identification, assessment, installation, maintenance/monitoring, and review/removal.

### Perceived impact of home sensors in social care

***Supporting independence and safety.*** It was highlighted that home sensors can support independent living in a safe environment in a way that helps maintain service user privacy. However, this relied on good knowledge of the individual’s existing routines, and in some cases, additional insight from the activity data introduced new concerns and perceptions of risk.

Staff, family/informal carers, and service users described how home sensors helped identify inactivity periods and reducing 'long lies’ after a fall, as well as unsafe ‘wandering’ outdoors. Sensors have also been reported to support activities of daily living, such as medication adherence, home temperature management, nutrition, and hydration, thereby reducing the need for frequent supervision.

*So if their room falls below a certain temperature, again an alert can go through to let somebody or their family know that that they turn their heating off. So we're preventing those, you know, hyperthermia and things like that.”* [Care Practitioner, Eastvale, CP_2]

Relatedly, many of the staff, family/informal carers, and service users interviewed felt this supported service user autonomy and dignity at home, reducing the need for formal and family/informal carer visits. One informal carer talked about how the sensors enabled her father, who refused all domiciliary support, to live alone despite his rapidly deteriorating dementia:

*“He's still got his personal life, living on his own, doing his own little weird work in his own little weird world...”* [Family/informal carer, Steelgate, S_18]

However, this did require careful and individualized trade-offs between support for autonomy and protection from harm. As illustrated in the below extract from the son of one service user, in which access to the activity data raised additional concerns about opening the front door, which he felt a need to restrict:

*“A couple of times she has had an unhealthy habit - I noticed the door was open for three or four minutes. And then it closes. So I rang her and asked her if she opened the front door – and she said ‘oh yes I did, just to see who was passing...’. And I was like ‘mum, you should not be doing that. You don’t know who is going passed your house’. She is lonely, that’s the problem.“ [Family/informal carer, Eastvale, FI_1]*

***Family/Informal carer reassurance.*** The use of home sensors was generally seen to reassure family/informal caregivers and reduce the burden of frequent visits or contact. However, this still required efforts among informal networks to monitor and respond to activity, effectively reshaping rather than merely replacing or reducing their caring roles and responsibilities.

Staff and family members across sites highlighted the value of being able to see the activity data, especially when the service user was not communicating (e.g. not answering the phone):

*It reassures me that my mother is either in bed or in her chair. I always check before I go to bed at night. I check she is in her chair, or bed, though she does not go in bed these days. And the front door is not open. … It does reassure me when I see her going from one room to another. I know everything is ok. I can breathe easy, until I look at the app again [Family/informal carer, Eastvale, FI_1]*

Within Riverbourne and Steelgate, service users were further reassured that there was a service monitoring their family members when they were unable to and would provide alerts of any problems identified by the sensors. For some families, a professional monitoring service was crucial, as they were already under strain to support the service user and were unable to constantly monitor the sensors.

However, this idea was queried by one family member who felt that no care provider or monitoring hub could know her mum like she could, and gave an example of a time her mum did something ‘normal’ (left the house during the afternoon) which was in fact highly unusual in its context and necessitated an emergency escalation:

*“It's not that they don't understand her or her situation or her motivations, just not in the same way that I do.” [Family/informal carer, Steelgate, S_20]*

Some family/informal carers noted that home sensors did not necessarily lead to less informal caring work; rather, the nature of the caring work *changes*.

*“It has eased the burden of needing to make routine phone calls literally just with the agenda of checking that she's OK, and has given time to get on with some of the more complicated things that we do - for housing, for coordinating her medical appointments, and those sorts of things and sorting out transport. [...] I've now got more time for dealing with that kind of stuff.”* [Family/informal carer, Riverbourne F_10]

Another aspect of reassurance for family/informal carers related to monitoring the activity of others visiting the person in their home (e.g. that domiciliary carers were visiting within the expected timeframes):

*“It kind of helps me see how long they are spending in the kitchen. I can’t see what they are doing in there, but can see how long. And often it can be very quick, the dishes can be very dirty. So it does give me that freedom to ring up head office and say this is happening and I am not happy with that. So the sensors in a way help cater for things that are not going so well. At least you have an idea of who is in which room and how long.” [Family/informal carer, Eastvale FI_2]*

***Identifying health care needs.*** Staff and family/informal carers provided examples of when the activity data informed health-related decisions, either from insights picked up by the formal monitoring services or based on their own interpretation of the activity data. However, such use of home sensors relied heavily on informal networks to take these insights forward with the relevant healthcare service. The extent to which these insights contributed to meaningful health outcomes and decisions was also unclear.

There were examples of home sensors being used to inform healthcare decisions. Common aspects of activity included increased bathroom usage (as an indication of UTI), nighttime agitation and wake-up patterns (as an indication of potential sleep disorders), and reduction in eating activities (as an indication of ill health or low mood). Within Riverbourne and Steelgate, such monitoring was undertaken as part of the short- and long-term care pathways. If changes were identified that indicated a need for action (referred broadly as ‘actionable insights’), then the relevant contact (e.g., family/informal carer, social worker) would be notified to take forward. For example, in Riverbourne, one family caregiver described how such data were used to request a sleep study from her son’s specialist health professional to investigate potential sleep apnea:

*“It's helped us to then corroborate what [service user] was saying about he didn't sleep well. And as a result of that, I took the graphs that they [social care services] sent me to the endocrinologist and said has he got sleep apnoea and can we investigate this? So that was investigated as a result of the extra evidence that we were getting from the monitors to say that they had a very unusual sleeping pattern.” [Family/informal carer, Riverbourne, F_2].*

However, as highlighted by one Steelgate staff member involved in remote monitoring, not only does such use of the technology require capacity to monitor the data, it depends on there being an individual able and willing to take forward:

*“You know, we can catch things early, but you have to be plugged in. You have to be interested in taking the steps.” [Staff, Steelgate, S_5].*

***More holistic and objective assessment for personalised care.*** It was highlighted that an analysis of service users’ activity patterns can be used to provide a richer and more objective account of their lives, capabilities, and support needs. However, this also raised challenges and uncertainties regarding how and the extent to which such quantitative representations should be relied upon against more direct meetings and encounters.

Social care practitioners discussed how activity data helped gain a fuller picture of an individual's life, especially for those unable to communicate their needs, and identify behavioral patterns and exceptions that might otherwise be unknown. Furthermore, it was reported that the data helped increase practitioner confidence in their assessments:

“ *To have a very evidence-based and informed level of assessment of need, and I think that's something that we've always found particularly difficult in social care.” [TEC practitioner/lead, Eastvale TPL_6]*

Additionally, home sensors were described as helping identify *‘points of escalation’* that, if acted upon, could help avoid unnecessary care placements, need for higher levels of care, and hospital admissions:

*“[it’s about] making sure that care is appropriate for that person, making sure people can live independently, safe and well, first and foremost. And that they receive the least restrictive care, so they haven't got people traipsing in and out of their home every day of the week - they get the right care at the right time when they need it.” [Staff, Steelgate, S_1]*

Examples were also provided as to how home sensor data were used to adapt and tailor a person’s care provision (e.g., decreased assistance with meal times, as sensor data showed that the service user could prepare their own food if encouraged and prompted earlier in the day).

However, it was also acknowledged by practitioners that *“you still have to sort of unpick it and do more sort of fact finding - it just gives you a start really to find out what is happening with people” [Staff, Steelgate_S_5]*. Many practitioners cautioned against placing too much weight on the sensor data, seeing it as something to be used in conjunction with existing assessment practices.

Similarly, some family/informal caregivers raised concerns regarding the extent to which the data would inform an assessment. The following extract is from one case in which the family felt that the home sensor data misleadingly downplayed one practitioner’s assessment of the safety concerns of their grandmother’s wandering:

*“I think that has caused me a lot of distress to be honest with you. […] We didn't always necessarily agree with the decisions of social care and like how they were using the data. [...] they, kind of, prioritized the data over other things.” [Family/informal carer, Riverbourne, F_7].*

***Supporting dialogue and communication.*** Some incidents were described in which the activity data appeared to work as an effective communication and ‘evidencing’ tool when considering and agreeing to an appropriate level of care for a particular service user. This appeared to be used on an ad hoc, case-by-case basis as opposed to routine practice.

In some cases, TEC leads and care practitioners highlighted the value of using activity data as a communication tool with commissioned care providers regarding the provision of care packages. For example, a staff member in Riverbourne described a situation in which a paid care provider advocated for a service user’s care package to increase because they regularly did not go to bed until 2am. However, the sensor data showed the service user in bed at 11pm and did not move until the morning. As highlighted in the following extract from a TEC practitioner in Eastvale, such uses of the data or facilitation tool or ‘boundary object’ ^36^ supported dialogue and shared understanding:

*“Sometimes care providers will say, you know, we need more care because we're doing XY and Z and then when you actually look, they're not really. Like it happens occasionally, but you can see it's not happened regularly. So then you might look at it together and say, well, this is what I'm seeing. It's, you know, it's different from what you're saying. How do we account for it? So you might use it in that way to have that conversation.” [TEC practitioner/lead, Eastvale, TP_4]*

Some practitioners also described how the data was used to ‘evidence’ additional care needs for internal reviews and decision making, such as special funding panels:

“ *If you go to a high cost panel and they say, OK, where's your evidence that the situation has changed, I personally would feel kind of very insecure about not having sensor data to back that up [TEC practitioner/lead, Eastvale, TP_3]*

An additional, and perhaps unintended, use of the technology by social care staff has also been to monitor care staff activity, cross-referencing care logs with sensor data where there have been care discrepancies or differing accounts from family members.

*"It gave us an idea of what's actually going on in that person's home while they're receiving this care. Because although we're commissioning it, this person is vulnerable, living alone, has dementia, we don't know what's going on in that environment. So it gave us a very good overview of what's going on. [...] (That sort of thing) happens more than I’d like to say!” [Care practitioner, Riverbourne, P_1].*

While some staff members considered the use of the technology to enable quality and safeguarding, others highlighted concerns and unease among some home care staff about their privacy and how the data would be used.

### Influences on the embedding and use of home sensors

***Materiality and dependability of the technology.*** Participants considered the technology generally reliable as long as there was adequate maintenance and connectivity, which could depend on both technological factors and user behaviors. Materiality (material properties, design features, and affordances) also influenced how and the extent to which it would fit within people’s lives and home environment. Effective use and sustained adoption required adaptive and practical efforts to ensure that the technology remained in good working order in people’s homes.

Across all sites, Wi-Fi connectivity was provided to service users who did not have broadband in place (at the cost of the council). Because of the typical user populations, this was a common requirement. In Steelgate, over 60% of service users required Internet access, which had not been factored into the initial phase of service provision. In some regions, particularly the rural areas of Riverbourne and Eastvale, the installers could not establish an internet connection, and thus the technology offered had to be withdrawn. Similarly, connectivity was sometimes influenced by building architecture (e.g., size, layout, and thickness of walls), resulting in poor internet signals that required installers to find workarounds:

*If there's a big house, if you've got like somebody with a really big house, we would provide them with two [internet routers]….With these little cottages that have got these very thick walls and that are really old we do have problems with that. But we have sort of like you put them on a windowsill.”* [Installer, Eastvale,TECP_2].

Staff and family/informal carers also talked about the practicalities of replacing batteries that had run out-of-charge. Across all sites, family/informal carers were shown or provided instructions on how to replace batteries during installation, and most said they were confident in doing so. However, maintenance support was sometimes required to replace batteries. In Riverbourne, the service delivery team incorporated routine maintenance visits (every six months) to people’s homes because the battery levels could not be monitored remotely.

Informal/family caregivers also mentioned situations in which sensors were displaced or fell from the walls, requiring either them or someone else to reattach them. As noted by the domiciliary carer below, engaging in such repair and maintenance work brought new risks and accountabilities:

*“I can put them back up, but then I don't know if they're pointing in the right place to sense (service user name) so it's easier if they (service provider) come and do it. And then they test it to make sure that it's working.” [Service delivery partner, Riverbourne, SDP_2].*

The technology might also be disconnected by the service users themselves or other people in their homes (e.g., unplugging the hub unit, removing sensor devices), requiring family members to recover and reconnect the devices, contact others to help them, or request a maintenance visit from the service provider.

*“But we're sort of looking on the back end as well. If [the sensors] are offline, if the battery's gone out of, they need new batteries, then we contact the person… So we keep an eye on things like. And then if we can't get in contact with responders or family, then we go out and do that tech visit, get it back online, replace the batteries. So there's always, you know, if the family aren't there and not prepared to manage it. We will go out and do it.”* [Installer, Eastvale, TECP_2]

***Compatibility of technology with service users’ lives and care networks.*** The use of home sensors works well and was considered useful to the extent that it aligned with the service user’s wider care network, priority concerns, and values. Staff and family/informal caregivers reported that service users’ initial responses to technology were mixed. Often, it was considered that the service user had forgotten about or did not notice the technology because of their cognitive impairment. In some cases, families needed to provide reassurance and familiarization, such as relating the technology with something that they knew the service user would understand or would be more palatable to them. Therefore, the introduction and explanations were a situated judgement based on the knowledge of the service user:

“ *Because of her dementia, we can't really explain to her what it is actually there to do, like actual purpose of it, because it would probably frustrate her. And, you know, she will find a bit confusing. So we just advised her that they're kind of alarms, you know, alarms for the property.”* [Family/informal caregiver, Riverbourne, F_7].

The balance between autonomy, safety, and dignity also necessitates various forms of ‘emotional labor’ ^37^ by members of informal and formal care networks:

*“They're [service user] like, ‘that's a camera, that's a camera’, and we have to really, you know, stipulate ‘this is not a camera this is just to help your family watch where you are, you know, if you do go to bed in the evening or you've not had a fall in a room, it is monitoring your movements..’ But most people are fine with it going in. Obviously social workers have spoken to them and have done their part. But yeah, it's sometimes challenging. Some people just don't want it. They're like, ‘no, take it away. I don't want it’.” [Installer, Eastvale, TECP_2]*

However, there were also occasions in which service users considered the technology too intrusive and had to be withdrawn. It was reported that the physical resemblance of some sensors to cameras increased such concern, and a sense of being ‘watched’. Some practitioners also reported that, for some service users, particularly within mental health services, the sensors were not conducive to their well-being, as it would heighten anxieties and paranoia.

The effective use of home sensors relied heavily on the involvement and engagement of informal carers to monitor and respond to data and alerts. For example, in Riverbourne, the proactive monitoring team would alert family members responsible for checking in or arranging for someone else (e.g., a paid carer or neighbor) to do so. In Eastvale, the long-term use of home sensors depended entirely on informal support. Similar concerns were raised regarding loss of engagement due to caregiver breakdown and difficulties maintaining the level of support and involvement required:

*“Whether or not the commitment is long term cause one of my biggest fears is we put something in place and we think this is going to be brilliant and then the family, you know, have carer breakdown, for example, and then a month in a row they can no longer hold up the obligations that they said they were.”* [TEC practitioner/lead, Eastvale, TP_4].

Across all sites, staff emphasized the importance of ‘wrap around’ support for the family/informal network, to align it with particular setup, capabilities, and priority concerns. The installers, for example, would often spend time with the family to help download the app on their phone, demonstrate how the sensor technology worked, and configure the devices and settings accordingly. In some cases, family/informal networks would adapt the technology, or request additions or adaptations to be made, once they become accustomed to the technology (and limitations) while in use. For example, there were a number of cases in which family members used home sensor data alongside video cameras to help contextualize and interpret the graphical visualization on the dashboard. This was explained by family members as a step towards comprising a more ‘tailored’ suite of technology, which provided a fuller picture of what was going on in the home; cameras were usually not in bedrooms or bathrooms. However, it was also acknowledged by some that the service users’ understanding of the placement and usage of cameras might be limited:

*“I've put ring monitor cameras in there so I can see what she's doing and bless her would be mortified if she knew...well we have told her, but I don't think she realises that they are cameras that you can dial in and see what she’s up to..” [Family/informal carer, Steelgate_S19]*

***Workforce knowledge, capabilities and capacity.*** Strategic decisions about the use of home sensors were generally made by TEC leads within the councils. However, the attitudes, capabilities, and actions of frontline social care practitioners greatly influenced the assimilation, embedding, and ongoing use of the technology. There were various aspects to this, including identifying service users who may benefit from the technology, tacit (know-how) knowledge of the ways in which the technology can be configured to meet particular needs, and interpretation of the sensor data. It was reported that social care practitioners varied both in their attitudes towards the use of home sensors and in their confidence and capabilities in applying the technology and interpreting activity data.

*“There is an issue with practitioners having the confidence, having the skill, whatever it is, to actually - not only put the sensors in, so identifying the cases where the sensors could be beneficial is one thing….Then the second thing is then actually using that data correctly and appropriately to then inform and evidence the judgments they need to make.”* [TEC practitioner/lead, Eastvale, TP_3].

Training and upskilling among care practitioners was generally done on the job as part of a team, as opposed to formal teaching or study. This was supported through multiple means across sites, including the establishment of local champions to advocate and support the use of the technology and provide a conduit between strategic decision-making and frontline practice. Members of the TEC teams or service delivery partners would also work closely with such champions to offer ‘drop-in’ sessions, informal training, and the development of internal guidance materials. Such interactions were reported as important for building workforce knowledge and confidence, as well as gaining insights into common problems or misunderstandings on the ground for wider learning and system change.

However, given their informal nature, such networks were also considered difficult to establish and maintain universally across counties, with varying levels of engagement. Some strategic leads within the TEC teams felt this was reflected in a higher proportion of referrals from ‘usual’ practitioners and early adopting regions and teams, as opposed to service user need:

*"User demographic is more about who is making the referrals (social care practitioners) rather than who can benefit, because the programme grew organically rather than to a plan”* [TEC team member, Riverbourne, ASC_1].

Variations in levels of engagement were linked to differences in practitioner attitudes, professional values, and styles of practice. The importance of focusing on positive messaging and cultural changes within the workforce has been highlighted by many strategic leads and champions. However, engagement among frontline practitioners remains a challenge in the face of limited time and capacity. Concerns about the extra logistical and administrative work that may be created by the use of home sensors further accentuated this challenge, particularly if the immediate benefits to staff or service users were not clearly evidenced or understood.

In addition to social care practitioners, domiciliary care workers were considered by TEC leads to be important for the success of sensor-based technology because of the frequent contact they have with the service user in their home; for instance, ensuring that the sensors remain online. This aspect of the care workforce tended not to be involved in the onboarding process and could be unaware of the purpose of the sensors and how they work, or concerned that the sensors may be monitoring their own activities and delivery on responsibilities. The high volume and heterogeneity of care, as well as variations in the level of domiciliary care provided to service users, made it difficult to operationalize enrolment and capacity building within this aspect of the care workforce.

***Inter-organisational routines and coordination.*** The sustained adoption and embedding of home sensors within the social care ecosystem required close attention to the co-adaptation of interdependent roles and workflows within and across organizations.

The staff emphasized the importance of clearly defined processes for home-sensor-enabled pathway delivery and differentiation in roles (advanced division of labor with different specialist functions allocated to different teams). For example, in Eastvale and Riverbourne, practitioners noted the value of having distinct responsibilities between social care practitioners (for assessments and referrals) and installers (for set up and continued support):

*“You've got people that are trained to know how to set them up… they can put them in the right place and make sure that they're working and whatnot. So that just makes it a bit easier and I think it makes people more likely to refer for [home sensors] because they know that there's somebody that's skilled and knows how to actually install whatever it is that we're looking to put in. It's very important to have like a dedicated team almost to be able to do that."* [Care Practitioner, Eastvale, CP_6]

The coordination of interdependent routines required the mutual adaptation of social and technical sub-stems across professional and organizational boundaries, supported by the joint and iterative development of 'structuring devices' ^38^ that guides and interconnects routine workflows. For example, in Eastvale, the TEC assessment form (used for ordering home sensors) was eventually integrated into the pre-existing social care assessment form hosted on the social care electronic record system, in order to effectively embed it within existing practitioner routines. This was subsequently linked to the technology providers CPR (Customer Reporting Management) system, enabling referrals to be sent directly on for installation, and in turn, keep referring practitioners updated as to when the technology had been installed, facilitating ‘mutual awareness’ (the sense of what the other collaborators are doing in order to provide a context for your own activity) ^39^.

In cases where technical integration was not possible (e.g., due to governance or interoperability constraints), staff would be required to bridge such systems as part of routine practice. For instance, in the case of Riverbourne, the home sensor dashboard and service delivery team IT systems were not integrated with social care IT systems; thus, referrals and PDFs of the dashboard activity data were securely emailed to the social care practitioners to manually save onto the social care record system. In Eastvale, lack of integration between NHS and social care systems meant the creation of dedicated staff within the hospitals to relay referrals onto the social care IT systems as part of the reablement and hospital discharge pathway.

The effective and continual co-adaptation of organizational routines and structuring devices over disparate systems relied on good collaborative relationships between the service delivery team, commissioning team, and technology provider. Establishing these processes was an ongoing accomplishment and remained a challenge in some key aspects of service delivery. In particular, structuring clear thresholds and protocols for proactive responses to activity data remained a persistent challenge. Even within Riverbourne, where a proactive monitoring team had been established, uncertainties remained with regard to organizational roles and accountabilities in operationalizing ‘actionable insights’ on a continual basis:

*"For short-term reablement, you need someone looking at the data consistently and regularly, and that isn’t really in place, and whilst the current service is probably compliant (as you don’t have to give them sensors) it’s a question of what if? that needs squaring."* [TEC team member, Riverbourne, ASC_1]

***Strategic alignment, evidence and funding.*** Readiness and capacity for the introduction and expanded use of home sensors were strongly influenced by strategic alignment across organizations, in which such initiatives had been assessed by respective organizations (either as business plans or informally) and considered favorable with strong senior-level buy-in. A key challenge was establishing shared goals and evidencing outcomes related to different institutional logic and strategic priorities.

Strategic alignment and collaboration were important for extending, adapting, and experimenting with different technological options, service processes, and team roles. For example, Eastvale initially formed the hospital discharge pathway through temporary funding of a digital practice lead post to facilitate referrals as well as the dedicated installer reserved by the technology provider to ensure rapid deployment. Once the feasibility and outcomes of the service model had been shown, the temporary post was made permanent through NHS funding, and the technology provider decided to continue and expand the enrolment of installers specifically for the hospital discharge pathway [see Box 1]. As highlighted below, strategic collaboration and alignment for this initial (temporary, experimental) arrangement were contingent on both formal and informal relationships, trust, and shared strategic interests:

*“I feel if we didn't have such a strong working relationship with [technology provider], because they were not contractually obligated to kind of help with that - we could have just been told ‘No. sorry, that's not doable, we're meeting our KPIs, this is what you guys are paying us for...’ But yeah, I think that working relationship really helped…But I think given the number of referrals they saw coming from the hospital compared to everywhere else, they [technology provider] really saw the value.”* [TEC practitioner/lead, Eastvale, TP_4]

In addition to the importance of long-standing and trusting relationships across organizational boundaries, this example illuminates the importance of systems and appropriate skills in place to monitor and evaluate the impact of the innovation. Accordingly, strategic leads across sites also talked about difficulties aligning mutual interests and demonstrating benefits with other key parts of the social care and healthcare systems:

“ *Health should be involved more because the outcomes achieved are often for them too. But there needs to be a good, open, public, shared strategy with some shared funding."* [TEC team members, Riverbourne, ASC_1]

Additionally, some TEC leads felt that strategic alignment with private care services remained a challenge, in part because reduced in-person care provision contradicted the domiciliary care business model:

*“Care providers are not a juicy market for this stuff because in some respects they want to sell visits...we have been around this many, many times and we’re working with the families.”* [Staff, Steelgate, S_4]

Strong leadership and senior-level strategic support appeared to be positively associated with securing funding and slack resources ^32^ to expand and sustain the use of home sensors across social care pathways. A challenge in this regard has been to make the case to invest in new approaches to proactive care, in the face of wider financial constraints across health and social care. As a technology supplier noted in the following:

*"It's difficult with proactive and preventative care because so much resources being pumped into the problem we've already got. Nobody's got the time to think about what's the problem that we might have in a minute and actually preventing from we might have in a minute."* [Technology provider, Riverbourne, TECH_3]

***Economic feasibility analysis.*** Some staff talked to us about how the sensors saved costs for health and social care services by reducing care packages, delaying residential care placements, reducing ambulance call-outs, and avoiding hospital admissions. Economic data for this study was lacking. However, it was possible to obtain descriptive data on the characteristics of home sensor users (ie service users provided with home sensor technology) compared to home care users (ie service users not provided with home sensors).

In the dataset provided by Eastvale, sensor users tended to be slightly older, although almost half of the sensor and home care users were 85 or older. There was slightly less ethnic diversity in home sensor users than in those without sensors, although a large part of this could be due to the much lower proportion of sensor users for whom this information had not been provided or obtained. The average IMD score of the sensor users was slightly lower, indicating that they were less deprived, although the difference was small. Sensor users were more likely than home-care users to have autism, dementia, other neurological health conditions, and learning disability support.

One interesting observation from the data provided by Eastvale indicated that sensors were being used by service users with more costly care packages, which persisted even when controlling for the features of the care user in a Propensity Score Matching (PSM) model. This technique first creates a probability (the “propensity score”) that each care user would be given home sensors based on their observable characteristics, such as age, gender, and health condition, and then selects individuals to identify groups with and without home sensors whose needs are more equal than in the equivalent groups observed in the overall sample.

In the example below, the control variables explain £365.80 of the total cost of care in the full sample, but this increases to £724.66 when looking at a sample defined using PSM. This means that home sensor users were significantly more expensive than care users who were not provided with home sensors and implies that there is another variable, such as the complexity of care needs, that we were not able to identify from the data we were using in this estimation. See Table 3 for descriptive data and Table 4 for the PSM model.

**Table 3.** Descriptive statistics of cost of care at start of care package.

Row Labels	Average of Total Week Cost at Start	Max of Total Week Cost at Start	StdDev of Total Week Cost at Start
Service user with no sensors	£379.64	£52,771.53	£1,040.69
Service user with sensors	£1,062.98	£11,827.04	£1,445.20
**Grand Total**	**£418.71**	**£52,771.53**	**£1,079.52**

**Table 4.** Propensity score matching.

Sample	Treated	Controls	Difference	Standard Error	T-stat
Unmatched	£1,004.60	£365.80	£638.80	60.37	10.58
Average Treatment effect among treated	£1,004.60	£724.66	£279.94	83.35	3.36

The data provided by Riverbourne presented a demographic picture similar to that of Eastvale, in which more than half were over 85 years old and the majority (79.6%) lived alone. However, the classification system used for primary conditions was much more limited than that used in Eastvale. The three main reasons for installing motion sensors were a “decline in memory” (47.1%), “decline in health” (21.8%) and “decline in physical mobility” (19.1%).

Our experiences demonstrate that it is possible to use a combination of primary and secondary data to analyze the cost consequences of home sensors, but significant time and resources are required in a full study to overcome the challenges that we faced in this rapid evaluation context.

## Discussion

### Summary of key findings

This evaluation of the use of home sensors for proactive care generated several key findings. Across the three sites, home sensors were used across multiple care pathways and contexts, including assessment, reablement (to varying degrees) and long-term care pathways. The *perceived* value and impact of home sensors in social care were numerous: Home sensor activity data were widely considered beneficial for service user independence and safety; family/informal carer reassurance; identifying health and social care needs; providing more holistic and objective assessments for personalised care; and communication and ‘evidencing’ need. However, the findings also highlight the tensions, uncertainties, and conflicting views on the use of home sensors and how they can shape the quality of care. In particular, there were some concerns regarding the reliability and suitability of quantitative activity data for care assessments and how this could be appropriately balanced against more direct and contextual information about the service user and their support needs. Additionally, while technology can provide reassurance and peace of mind for family/informal carers, there is potential for unintended consequences of increasing caregiver anxieties and demands, especially if the technology is not supported or compatible with people’s needs, priorities, and capabilities.

Implementation and use varied across sites and were shaped and constrained by numerous factors, including technical, regulatory, social, financial, and logistical contingencies. The adoption and embedding of home sensors in routine practice was an ongoing pursuit across case sites involving incremental and distributed changes across a nexus of interconnected cross-sectoral systems and practices. Key areas of focus in this regard included the material properties and dependability of the technology (involving maintenance work and supporting infrastructures); compatibility with service users and their care networks (aligning use with needs and capabilities of service users and their care networks); workforce knowledge and engagement (e.g., confidence, skills, and capacity); inter-organizational routines and coordination (co-adapting routines and supporting structures across organizational boundaries); and strategic alignment and funding (including senior-level leadership and buy-in, collaborative partnerships, and slack resources for continual adaptation and experimentation).

Despite some promising insights into the potential benefits to service users, family/informal care, and health and social care services, there remains a lack of compelling evidence on system-level impacts and cost-effectiveness. This has been underpinned by challenges in collecting and accessing relevant data. Further work is needed to support local evaluation capabilities to monitor and assess the impact and cost-effectiveness in ways that continually inform strategic and operational decisions on the implementation and scaling up of home sensors for proactive care.

### Comparisons with previous literature

This study extends existing literature on the use of home sensors in social care. As reflected in our initial scoping literature review, participants described the multiple roles of such technology, including assessing care needs, supporting timely interventions, and providing reassurance. However, there is little published detail in relation to the on-the-ground experience of implementing, embedding, and using such technology within routine care practice, and implications for sustained adoption at scale. Our findings, therefore, provide further insight into experiential accounts with the technology and services around it, and the work of introducing and embedding such technology within social care.

Our findings illuminate how the effective use of home sensors emerges through situated practices and relational negotiations, ‘reshaping’ (as opposed to replacing) care roles and practices. For example, such technology introduces new and extended roles for family/informal care networks to monitor data and respond to associated insights. The emergent use of home sensor data by practitioners (e.g., to assess and evidence need or monitor care provision) also raises practical, interpersonal, and ethical implications regarding how such data are used. This reflects Pol’s ^40^ emphasis on the need to understand and accommodate the human and relational dimensions to technology-enabled care if it is to become successfully adopted and embedded within frontline care practice.

Additionally, following our initial scoping literature review, we specifically sought to shed light on the practical and logistical challenges of incorporating technology within social care workflows and infrastructure. Our findings in this regard resonate with wider socio-technical systems theory, which has long emphasized the need for mutual adaptation of technologies and processes to extend and improve the fit between them ^41, 42^. In particular, the case sites draw attention to the importance of attending to ‘organizational routines,’ defined as ‘recognizable, repetitive patterns of interdependent action carried out by multiple actors’ ^34^ across organizational and sectoral boundaries. Routines help reduce uncertainty, support complex collaborative work, and maintain ‘mutual awareness’ ^39^ of distributed roles and actions. Attending to routines is therefore important in establishing the foundations for implementation (through alignment with established routines and minimizing disruption to existing practice), scale-up (through stability and replicability), and sustainability (through consistency in process and retaining organizational memory). Organizational routines are also situated within a socio-material context; in that, actions are structured around time, physical spaces, material, and technological artifacts. Within our data, we observed the importance of co-adapting routines and associated ‘structuring devices’ (e.g. online forms, electronic record workflows, protocols) ^38^, in order to maintain clear processes and roles and accountabilities between staff. This required close collaborative work across the social care, service delivery, and technology provider teams, which has been key in this regard, requiring time and long-term strategic planning to build relationships and trust for ongoing adaptation and system learning. However, defining and negotiating such routines and accountabilities remained an ongoing challenge in many aspects of service delivery. In particular, uncertainties remained in the formalizing of decision-making protocols and accountability with regard to proactive responses to home sensor activity data.

Our focus on organizational routines further highlights the need to consider various types of ‘articulation work’ being performed by different actors involved in the provision, maintenance, and day-to-day management of home sensors. Articulation work can be described as a ‘supra type of work’ that connects and integrates tasks and ensures activities ‘mesh’ across divisions of labor ^43, 44^. In our study, we observed the role of articulation work in the coordination of activities (e.g. between referring practitioners and technology installers), data management (e.g. sharing and information across disparate IT systems), keeping the technology in working order (e.g. maintenance visits, check-in calls and monitoring technology status in use) and helping manage relationships (e.g. ‘emotional labor’ ^37^) with service users and their families. We also observed the contribution of articulation work undertaken by service users’ families and informal networks as they attempted to appropriate and ‘domesticate’ ^45^ the technology within the home, and take on extended roles in monitoring, responding, and interacting with call center operators, for the system to work as a whole. Previous research has shown how a mismatch between formal procedures and what is actually necessary in practice to ‘get the job done’ may compromise the scalability of the service at the organizational level ^44, 46^, stressing the importance of having mechanisms to help surface such work and establish how it may be supported and resourced efficiently, and hence more sustainable and scalable.

Our findings on workforce influence reflect wider debates on how ‘digital skills’ are defined and understood in health and social care ^47^. With regard to home sensing, social care practitioners’ knowledge was not limited to how the technology worked in general, but involved situated knowledge and skill, which extends beyond general awareness and understanding of the technology. There has been much policy interest in ensuring that the health and social care workforce has the right skills to apply and utilize digital technologies ^48^. However, as highlighted by Whitfield *et al.* ^49^, current policy discourse on skilling the workforce underplays the diversity of roles across the care system and overlooks the need to manage the heterogeneity and shortcomings of digital devices and systems adaptively. The adoption and use of home sensors across social care relied heavily on the development and sharing of tacit (know-how) knowledge through a combination of formal training (e.g. demonstrations with technology by providers), informal support (e.g. drop in sessions on activity data interpretation) and ‘learning by doing’ (e.g. how aspects of the technology can be configured for particular needs, troubleshooting issues) across multiple actors in the system. However, opportunities for learning and support were often limited to an already overstretched, under-resourced workforce and often constituted additional ‘hidden work’ that was difficult to manage and sustain.

In addition to understanding the process of implementing and embedding the use of home sensors in social care, we also sought to assess service-level outcomes through economic analysis. As highlighted in the initial scoping review, while there are widely held perceptions and assumptions on the cost benefits for health and social care, there is currently a lack of methodologically robust evidence. Our collaborative efforts on this component revealed how the national absence of such evidence is not merely a methodological issue but is underpinned by wider infrastructural (technical and regulatory) challenges faced widely across the social care sector. Similarly, Mendes *et al.* ^50^ recently highlighted how system fragmentation significantly limits the scope of comprehensive economic analysis across health and social care, linked to data access and governance constraints. Improved integration is needed to gain a deeper understanding of how and the extent to which home sensors can advance proactive care and mobilize resources and investments to scale up and spread such service models. This calls for closer engagement at the national policy level, in collaboration with regional stakeholders across health and social care. We have found that domains to focus on include service set-up and running costs (including technology, supporting infrastructure, service delivery and ‘wrap around’ support), social care provision (including other technology as well as in-person care), health outcomes (including hospital admission and treatment), as well as social and quality of life data (e.g. using short standardized measures within assessments and reviews).

Finally, our analysis affirms Glasby *et al.*’s ^3^ previous study of similar technology, highlighting that existing pressures on health and social care constrain the scope of pursuing a wider strategic interest in the long-term benefits of proactive and preventative approaches to technology-enabled care. Our findings are in line with the wider literature on the diffusion of innovations that emphasizes the importance of establishing shared (and evolving) meanings and values in relation to the innovation in use, and a coherent and shared understanding or ‘organizing vision’ ^51^ for describing the role and impact.

### Recommendations

The findings offer key insights and recommendations relevant to different stakeholder groups.

**For national policymakers:**

- **Work with cross-sectoral professional networks to co-create guidance and collaboration on service change.** Various related forms of guidance have, or are, being developed that may inform the implementation and use of home sensors (e.g. the TSA’s ‘blue print’ for proactive and preventative care services ^52^ and the DHSC’s articulation of "What Good Looks Like" for technology in social care ^53^). Collaborative consolidation and alignment across emerging resources and expertise would help form coherent national-level technology-agnostic guidance. Such resources should incorporate a complexity-informed framework to iteratively guide operational decisions for organizations at different stages and levels of maturity. For example, the NASSS Complexity Assessment Tool (NASSS CAT) is an instrument that builds on NASSS domains to guide technology-supported change programs ^54^. This could be further supplemented through the use of quality improvement collaboratives to maximize inter-organizational learning at the national and local strategic levels.
- **Look to integrate digital competencies within social care education and continued professional development pathways:** Progress has been made in this area at a national policy level, such as through the DHSC Digitising Social Care Programme’s Digital Skills framework ^48^. However, there is scope to continue working with relevant educational and regulatory bodies to establish potential training pathways, certifications, and incentive structures to advance skills and expertise in TEC provision, acknowledging the diversity in roles at both the strategic and frontline levels of the workforce.
- **Develop a minimum dataset framework for evaluation.** A nationally structured set of metrics outlining essential data elements (financial, health, and quality of life) and appropriate sampling (e.g., by including a control group) will assist local authorities in managing and standardizing approaches to evaluate the impact and value of home sensors for proactive care. Such a framework should seek to leverage existing routine data where possible, with a focus on demonstrating changes in outcomes for systems and service users and the associated cost consequences. Our findings indicate the need for a holistic approach across multiple interacting domains, including care, social, and process-related outcomes.
- **Invest in infrastructure and local capacity for evaluation.** Attention needs to be paid to the technical and regulatory constraints to harness data for continual, robust evaluation of the impact and cost-effectiveness of home sensors, and crucially, to feed into the business case for longer-term provision of such approaches to remote monitoring (beyond the pilot). This includes data linkages across health and social care as well as other sectors constituting service delivery. Recent progress has been made within the residential care home setting (e.g., the DACHA study ^55^), from which transferable lessons may be drawn for the domiciliary context (e.g., clarity on data sharing and streamlining information governance). Key areas identified in our study include the need for clarity and consistency regarding service user consent to use personal data for evaluation purposes, de-identification and anonymization requirements, data security specifications, and agreeing arrangements on data ownership and control.

**For local authority decision makers and commissioners:**

- **Enable and support incremental approaches to innovation and service development.** The technology cannot be merely installed or deployed at scale, but instead needs to be organically ‘grown’ in a way that takes account of changing organizational needs and existing systems, resources, and processes. This requires a judicious balance between top-down managerial (clear processes and milestones) and emergent bottom-up (responsive and contingent) approaches. Additionally, it requires ‘slack’ resources and funding (i.e., beyond minimal requirements to maintain operations) that can be channelled into new projects, experimentation, horizon scanning, and wider stakeholder engagement.
- **Establish evidence-based commissioning criteria and routes to guide implementation and decision making.** Ensure that decisions are driven and adapted according to data, user needs and experiences, and key outcomes linked to proactive care. Additionally, cross-sectoral strategic alignment and partnerships share the responsibility for delivering and evaluating outcomes.
- **Take a systemic and relational view of technologies (versus viewing it as an isolated tool or function).** It is important to consider the use of home sensors as part of a wider system of care. This includes attending to the ‘hidden work’ and unintended consequences of use that need to be addressed and adequately resourced. In particular, home sensors require adaptive and person-centered approaches to providing ‘wrap around’ support for service users and their networks of care.
- **Remember that technology-enabled care is cooperative and embedded within organisational routines.** Substantial work is needed to align processes within and across organizations that are already extremely busy and understaffed. Careful attention should be paid to how the logistical and infrastructural arrangements for home sensors align with the existing workflows and processes. Service delivery and monitoring processes also require a clear functional differentiation between staff roles and accountability. Particular attention is needed in relation to organizational roles and accountability for proactive monitoring and response.
- **Encourage and support practitioner engagement using ‘champions’ and ‘super users’ within peer support networks.** Continue to identify and systematically harness the energy and expertise of different ‘champions’ (advocates for the technology, promoting positive attitudes and influencing organizational culture) and ‘super-users’ (members of staff with deep knowledge and skills who can support other members of the organization). This could be facilitated through formalizing, recognizing, and rewarding such roles (e.g., through career development, time allocation, stipends) and creating structured opportunities for peer support and shared learning (e.g., drop-in sessions, shadowing, peer training). Key areas for consideration relate to the integration and configuration of home sensors within care practices, utilization of home activity data, and technical knowledge of the inner workings, capabilities, and limitations of home sensor technology.
- **Establish local evaluation roles, processes and systems for data management and analysis**. Significant resources are needed to continually manage, maintain, and analyze data related to the impact and outcomes of home sensors across different care pathways. This requires distinct internal roles and expertise to maintain and improve the quality of routine data (e.g., linking different data sources, cleaning, and validating the data), ensuring adequate data are routinely collected, reviewing data gaps and opportunities, and undertaking and sharing analyses for ongoing service development and strategic decision making.
- **Seek to harness and utilise data already collected as part of routine practice.** Routine administrative, health, and social care data for operational purposes offer potentially valuable data sources for evaluating service-level outcomes in terms of impact and cost-effectiveness. Attention needs to be paid to establishing how relevant data sources (across different systems and organizations) can be linked, ensuring that data collected at the frontline is adequate for evaluation purposes, and identifying opportunities or gaps where relevant data could be captured within frontline practices.

**For social care practitioners**

- **Work with service users and their care network to co-produce personalised and workable solutions**. Drawing on wider guiding frameworks on person-centered care, develop and share best practices in grounding the use of home sensors in an understanding of the needs and wishes of the service user and the family/informal carers. It is important to acknowledge how and the extent to which the technology fits within the wider network of care, including the capacity and capabilities of the informal support network and the need to monitor, adapt, and respond to changing needs and unintended consequences.
- **Build, support and maintain communities of practice within and beyond organisational boundaries.** Regular interaction and knowledge sharing through communities of practice will help foster learning and confidence building regarding the use of home sensors in care practice. However, by their very nature, communities of practice are informal and unstructured, and therefore require professional bodies and practitioners to take an active role in driving and sustaining meaningful and consistent communication and clear purpose, while encompassing psychological safety (e.g., feeling safe to share challenges, ethical dilemmas, and decisions without fear of judgement) and confidentially (maintaining trust and privacy).
- **Collaboratively produce guiding principles on the practical and ethical application of home sensors in social care.** Social care practitioners should work together alongside professional bodies to devise quality principles in relation to the rapidly emerging uses of home sensors in different care contexts (e.g., approaches to understanding/gaining consent, use of activity data to inform care assessments and provision decisions, and using activity data to monitor or cross-reference domiciliary care activities). Given the need for situated and adaptive approaches to such practices, it may be unwise to attempt standardized procedures. However, there is potential to offer ‘rule of thumb’ guidance to provide clear direction (e.g., using the Delphi method for structured and iterative consensus building around complex and contextually dependent care practices).
- **Engage in the contribution of ongoing development and evaluation.** Acknowledge the value and importance of feeding back real-world experiences in the provision and use of home sensors for wider strategic planning and decision making. In particular, seek to increase awareness and appreciation of the technical, social, and ethical complexities to be considered at a strategic level. Promote and engage in ‘reflection in action’ with regard to how use of the technology shapes professional caring practices and implications for service delivery and quality of care.

**For technology providers**

- **Attend to basic design properties and dependability of the technology.** Recognize how the subtle and often taken-for-granted aspects of the technology (e.g., appearance, aesthetics, usability, and connectivity) can have a significant impact on sustained adoption. Additionally, it is important to acknowledge the need to take active roles in providing ongoing maintenance and support as part of the care system.
- **Work closely with social care partners to continually co-adapt technologies and service processes.** Work collaboratively and adaptively within social care organizations to establish clear roles, processes, and accountabilities across diverse settings, with variations in digital maturity and expertise. Seek to make technical work more visible to nontechnical social staff (e.g., through demonstrations, accessible and clear guidance), allowing them to take a more informed leadership role in using and embedding technology within routine practice. In turn, it is important to become attuned to the nuances of technology use (e.g., shadowing and speaking with practitioners and service users), thereby understanding the importance (or not) of particular features or adaptations.
- **Work with social care organisations and related technology providers to facilitate system integration.** This includes technical challenges associated with the integration of activity data with relevant health and social care record systems as well as the scope to expand the use of home sensors alongside other TEC provider systems and devices.
- **Recognise the role and potential data points for evaluations.** Data captured by technology providers for administrative and operational purposes offers potential value for ongoing evaluation purposes. This could include, for example, the utilization of the technology and service, technical support requirements, incident reports, and technical set-up costs across service users. Therefore, it is important to work with social care organizations and relevant partners to establish data collection protocols and sharing agreements for evaluation.

In summary, sustained adoption of home sensors ‘beyond the pilot’ demands a coordinated, multi-level approach across all sectors and levels of care. This should include a focus on: consolidating emerging guidance and expertise; investment in workforce capacity and skills; evidence-based commissioning for incremental and iterative service development; support and guiding frameworks for local evaluation capabilities; and cross-sectoral partnerships to continually co-evolve the social and technical subsystems that constitute service delivery.

### Strengths and limitations

This rapid evaluation is small-scale and was conducted over a period of 13 months, during which data availability and access needed to be established and agreed upon. Available data for the economic component were limited; therefore, a comprehensive analysis could not be conducted. However, the process of assessing data availability in collaboration with our site partners provides useful insights for methodological, practical, and policy recommendations for future economic analyses.

While the study generated rich qualitative data, a larger study with more in-depth and longitudinal data could have provided further insight into contextual influences and complexities and how these change over time. The findings are drawn on three diverse sites that are not generalizable to the wider social care sector. However, through a theoretically informed analysis alongside wider stakeholder engagement, we sought to draw transferable lessons to inform future policy, practice, and research. Through system mapping and strategic sampling, we interviewed a diverse sample of staff and system stakeholders as well as service users and family/informal carers. We did not gather data from people who had not been offered home sensors or who were unable to access the technology. Furthermore, we were unable to interview service users who lacked the capacity to consent due to governance restrictions for the service evaluation. Given the high proportion of service users with cognitive impairment, a longer study with relevant ethical approvals with MCA (Mental Capacity Act) consultee procedures could provide insight into the lived experience of this user group. However, our study significantly extends the existing body of literature with regard to lived experience and care practices related to the use of home sensors for proactive care.

## Conclusion

Home sensors present multiple impacts within the context of proactive care, including timely identification of situations where a service user may need intervention, assessing the nature of care that is needed, and providing reassurance to family/informal carers. While the use of home sensors for proactive care aligns well with wider policy and strategic aims for preventative care and digitization, persistent demands and capacity pressures often prioritize immediate and acute needs over proactive approaches. Investment and mobilization across stakeholders is also limited by the lack of evidence on the impact and cost-effectiveness for health and social care.

Our findings suggest a need to: acknowledge the labor-intensive process of embedding and adapting use of the technology and a need to innovate incrementally; attend to the materiality and dependability of the technology, and supporting maintenance roles and structures; develop and resource tailored ‘wrap around’ support, in order to continually align, monitor and adapt solutions with services users and their networks of care; invest in multiple channels and networks to build workforce skills, interest and confidence; remember the importance of organization routines within and across organizational boundaries, with a particular focus on roles and accountabilities regarding proactive monitoring; invest in building evaluation capabilities and addressing data sharing and governance constraints; and promote cross-stakeholder learning and dialogue on the role and value of home sensors for proactive monitoring to facilitate strategic alignment and collaboration.

## Consent

All service users and staff interviewed provided written informed consent, and all references to participant quotes were de-identified to protect anonymity.

## Data Availability

Raw data from this mainly qualitative study cannot be published due to ethical considerations, as some data may be personally identifiable. We are unable to share raw quantitative data as this was provided by participating sites under a data sharing agreement. Selected data will be made available to bona fide researchers with appropriate ethical approvals on reasonable request to the lead author (email: joseph.wherton@phc.ox.ac.uk). Open Science Framework (OSF): Adoption and spread of technology-enabled home sensors in social care. Includes case site summaries (pseudonymised) for Riverbourne, Steelgate and Eastvale. DOI: https://osf.io/586j7/ ^56^. Extended data are available under the term of the Creative Commons Attribution 4.0 International Public License (CC BY 4.0 license).
