# Supplementary material for: Adopting and embedding home sensors in social care: findings from a mixed methods, rapid evaluation
Source: NIHR Open Res. 2025 Oct 6;5:71. Originally published 2025 Aug 22. [Version 2] doi: 10.3310/nihropenres.14011.2 (PMC12511854; doi:10.3310/nihropenres.14011.2)
Supplement: Peer review history

# Reviewer report: Reviewer response for version 2

**DOI:** 10.3310/nihropenres.15364.r37560

**Published:** 2025-10-09

**Authors:** Carl A. Thompson

**Affiliations:** 1 University of Leeds, Leeds, England, UK

The team have done a good job in addressing my comments - well done.

Is the work clearly and accurately presented and does it cite the current literature?

Yes

If applicable, is the statistical analysis and its interpretation appropriate?

Partly

Are all the source data underlying the results available to ensure full reproducibility?

No source data required

Is the study design appropriate and is the work technically sound?

Yes

Are the conclusions drawn adequately supported by the results?

Yes

Are sufficient details of methods and analysis provided to allow replication by others?

Partly

Reviewer Expertise:

Implementation Science, Social Care, Evaluation of Technology

I confirm that I have read this submission and believe that I have an appropriate level of expertise to confirm that it is of an acceptable scientific standard.

# Reviewer report: Reviewer response for version 1

**DOI:** 10.3310/nihropenres.15234.r36990

**Published:** 2025-09-12

**Authors:** Grant Gibson

**Affiliations:** 1 University of Stirling, Stirling, UK

This paper provides a detailed account of a rapid evaluation of technology enabled care services within social care, making use of increasingly prevalent range of home sensors within existing technology enabled care services. I felt that this report makes an extremely valuable contribution to the wider literature on technology enabled care services more widely, and specifically for proactive forms of telecare using complex arrays of home sensors for detailed activity and health monitoring of residents.  

 The main strength of the report is its detailed, holistic examination using the NASSS framework to conduct a whole system evaluation of the chosen three studies. The NASSS, developed by members of the team for this project has demonstrated itself to be a highly valuable tool for holistic, embedded evaluations of Technology enabled social care services, and the team once again demonstrates its relevance in this space. The authors have collected a large amount of qualitative data across the three local authority services, with data being collected across the various staff members, stakeholders within related services, and service users and their carers. Key findings include the paper's demonstrating of the need for consideration of issues affecting implementation and adoption of sensor across the health and social care spaces, including the detailed and often hidden work that stakeholders involved in delivery (Staff, service users and carers) have to put in place - in doing so the paper demonstrates the multiple real world actors and actants that are required to make home sensor systems 'work', and the various challenges that can influence their adoption. The report makes a useful contribution in identifying what some of these local variables might be, providing insights that can be applied across social care services at scale, even if the practical solutions may differ to each service.

 The paper also identifies key challenges and gaps that limit our ability to generate evidence about the effectiveness of these technologies. These gaps have been identified before, however this report significantly adds to the recognition of gaps in available evidence, specifically in relation to gaps within the data generated within services themselves and gaps in relation to economic assessment of services. These gaps pose significant limitations on our ability to generate a strong evidence base and the report makes an important contribution to further signaling these gaps, as well as providing recommendations regarding how data can be collected within and by services to help fill this gap.

 The paper concludes with a number of highly relevant recommendations both for practice, policy, technology design and research, all of which will hopefully make a significant contribution to the future research.

 In terms of issues I had few.  There is certainly an opportunity for further in-depth discussion of some of the extensive qualitative data generated in the project as mapped onto the NASSS. I would also be very interested in a methodological discussion and reflections regarding the process of navigating the various gatekeepers and partners within the three case study services as I expect this would pose numerous challenges - some more information of these challenges could be provided and would be extremely useful for future researchers to read. The economic evaluation was limited as the authors recognize, I would like more information about the Propensity Score Matching approach adopted as the discussion of this and its relevance was relatively brief although i accept that the data available to make a detailed economic assessment was limited, and this is an important insight for future research and for future service development.

 But overall I feel this paper makes a significant and valuable contribution to the literature on technology enabled care, makes a strong demonstration of the utility of the NASSS as a means to conduct detailed, holistic and multi-domain analyses of factors influencing the success (or not ) of telecare services.

Is the work clearly and accurately presented and does it cite the current literature?

Yes

If applicable, is the statistical analysis and its interpretation appropriate?

I cannot comment. A qualified statistician is required.

Are all the source data underlying the results available to ensure full reproducibility?

Partly

Is the study design appropriate and is the work technically sound?

Yes

Are the conclusions drawn adequately supported by the results?

Yes

Are sufficient details of methods and analysis provided to allow replication by others?

Yes

Reviewer Expertise:

Technology enabled care.  Social Care. Qualitative methods in social care research.

I confirm that I have read this submission and believe that I have an appropriate level of expertise to confirm that it is of an acceptable scientific standard.

# Response

**Published:** 2025-09-26

**Authors:** Joseph Wherton

**Affiliations:** University of Oxford, Oxford, England, UK

**Reviewer 2:**

*This paper provides a detailed account of a rapid evaluation of technology enabled care services within social care, making use of increasingly prevalent range of home sensors within existing technology enabled care services. I felt that this report makes an extremely valuable contribution to the wider literature on technology enabled care services more widely, and specifically for proactive forms of telecare using complex arrays of home sensors for detailed activity and health monitoring of residents.*

**Response:** Many thanks

*There is certainly an opportunity for further in-depth discussion of some of the extensive qualitative data generated in the project as mapped onto the NASSS.*

**Response: **Thank you - we completely agree that there is much more to explore within the qualitative data. Our aim with this paper was to provide a comprehensive overview of the key findings related to the main evaluation questions. Additional papers are currently in preparation, offering more focused accounts that build on the key themes.

*I would also be very interested in a methodological discussion and reflections regarding the process of navigating the various gatekeepers and partners within the three case study services as I expect this would pose numerous challenges – some more information of these challenges could be provided and would be extremely useful for future researchers to read.*

**Response: **We have added text in the *strengths and limitations* section of the *Discussion* to include reflections on the practical and methodology challenges of navigating the care services. In particular, we have expanded on the role of gatekeepers, and methodological implications within the rapid evaluation context.    

*The economic evaluation was limited as the authors recognize, I would like more information about the Propensity Score Matching approach adopted as the discussion of this and its relevance was relatively brief although I accept that the data available to make a detailed economic assessment was limited, and this is an important insight for future research and for future service development.*

**Response: **We have now added further information about the PSM method in the paper and in response to the questions asked by reviewer 1 above.

# Reviewer report: Reviewer response for version 1

**DOI:** 10.3310/nihropenres.15234.r36994

**Published:** 2025-09-04

**Authors:** Carl A. Thompson

**Affiliations:** 1 University of Leeds, Leeds, England, UK

General Comments

 The team have presented a timely and relevant (to policy, academia and practice) evaluation of home sensor technologies in adult social care across three local authority sites. The study is methodologically sound, theoretically grounded, and offers valuable insights into the challenges and enablers of technology adoption in complex care environments.

 The use of the NASSS framework, supplemented by the additional three theoretical "lenses", makes for a solid and appropriate conceptual foundation.

 Reporting is clear and (in the main) transparent. The discussion and treatment of limitations is thoughtful and sufficient in scope and depth.

 Major Comments:

 1.     Conceptual Integration:

 The manuscript would benefit from a clearer articulation of how the supplementary theoretical frameworks (diffusion of innovation, organizational routines, information infrastructure) complement the NASSS framework. Although not quite sure than the routines and the infrastructure sources represent "frameworks" or "theory" in the ways in which NASSS and DOInnov do. While these concepts are referenced, their analytical contribution could be more explicitly demonstrated throughout the findings.

 2.     Methodological Clarity:

 The qualitative sampling strategy (purposive and snowball) is appropriate, but further detail on how saturation or thematic sufficiency was assessed would strengthen the methodological transparency. Additionally, the role of gatekeepers in shaping participant recruitment warrants reflection.

 The qualitative analysis is rather scant and opaque (what does [NASSS] "used adaptively" mean?). Similarly, any triangulation details (between method etc) is lacking.

 Net result: "trustworthiness" of findings and conclusions is rather mitigated. Need more clarity and detail here. 

 3.     Economic Evaluation:

 The economic analysis is presented with appropriate caution. But Propensity Score Matching (PSM) methodology requires further clarification:

 •     which covariates were used in the matching process.

 •     how was balance assessed post-matching.

 •     were any sensitivity analyses conducted? Given the considerable uncertainty implied in the findings, this would seem appropriate. 

 Dealing with These would aid interpretation of the findings and inform future cost-effectiveness research.

 4.     Equity:

 The manuscript touches on digital exclusion and compatibility with service users’ lives, but a more detailed discussion of equity - especially with regard to under-served populations - would add value. Consider elaborating on how inclusion was addressed in the evaluation design and the implications for future deployments of the TEC.

 5.     Data Governance and Infrastructure:

 The challenges around data sharing and governance are well-documented. And there were obviously problems in accessing data for analysis. Giving space to a more critical reflection on the systemic implications of these barriers -particularly in relation to policy and LA infrastructure - would increase the manuscript’s contribution and doesn't depend on data (ironically).

 Minor Comments:

 •  Plain English Summary: Well-written; minor simplification of technical terms (e.g., “activity data”) may improve accessibility.

 Figures and Tables:

 •     Figure 2: Annotating with site-specific examples may enhance clarity.

 •     Tables 3 and 4: Add explanatory footnotes for abbreviations and statistical terms.

 •     References: Ensure consistency in formatting and inclusion of DOIs where applicable

 Strengths:

 •     Rich qualitative data from diverse stakeholders.

 •     Clear articulation of implementation challenges and enablers.

 •     Constructive recommendations tailored to different stakeholder groups.

 •     Transparent discussion of limitations, especially regarding economic evaluation.

 Recommendation:

 I think its suitable for indexing after (minor) revisions mainly to methods. The manuscript makes a valuable contribution to the literature on technology-enabled social care. It will  (should) interest to researchers, practitioners, and policymakers.

Is the work clearly and accurately presented and does it cite the current literature?

Yes

If applicable, is the statistical analysis and its interpretation appropriate?

Partly

Are all the source data underlying the results available to ensure full reproducibility?

No source data required

Is the study design appropriate and is the work technically sound?

Yes

Are the conclusions drawn adequately supported by the results?

Yes

Are sufficient details of methods and analysis provided to allow replication by others?

Partly

Reviewer Expertise:

Implementation Science, Social Care, Evaluation of Technology

I confirm that I have read this submission and believe that I have an appropriate level of expertise to confirm that it is of an acceptable scientific standard, however I have significant reservations, as outlined above.

# Response

**Published:** 2025-09-26

**Authors:** Joseph Wherton

**Affiliations:** University of Oxford, Oxford, England, UK

***Response to Reviewer 1***

 Many thanks for your review comments and suggestions. Please find our point-by-point response below.

*The team have presented a timely and relevant (to policy, academia and practice) evaluation of home sensor technologies in adult social care across three local authority sites. The study is methodologically sound, theoretically grounded, and offers valuable insights into the challenges and enablers of technology adoption in complex care environments. The use of the NASSS framework, supplemented by the additional three theoretical "lenses", makes for a solid and appropriate conceptual foundation. Reporting is clear and (in the main) transparent. The discussion and treatment of limitations is thoughtful and sufficient in scope and depth.*

**Response:** Thank you

*The manuscript would benefit from a clearer articulation of how the supplementary theoretical frameworks (diffusion of innovation, organizational routines, information infrastructure) complement the NASSS framework. Although not quite sure than the routines and the infrastructure sources represent "frameworks" or "theory" in the ways in which NASSS and DOInnov do. While these concepts are referenced, their analytical contribution could be more explicitly demonstrated throughout the findings.*

**Response:** We have added text to the *Methods* and *Findings* sections to better articulate links between the supplementary theoretical literature and the empirical data. The concept of information infrastructure helped deepen our theorisation of emerging finding related to the *technology* domain of the NASSS framework, with particular focus on the ongoing, and often-hidden, maintenance and repair work to support sustained adoption of the technology. We have included additional text within the sub-section on *materiality and dependability of the technology*, in order to highlight this link. The theory on organisational routines guided further analysis related to the NASSS domain on the *organisational *context *. *Specifically, theoretical literature on routine dynamics within organisational change guided our analysis of the interdependencies across social care workflows. These aspects of organisational routines acted as sources of inertia, unintended consequences and emergent change. They also helped illuminate the role of material artefacts and systems (e.g. assessment forms, social care record systems) and how these shaped, and were shaped by, the use of home sensors. We have added text to the relevant sub-section on *inter-organisational routines and co-ordination*, in order to highlight this. Finally, the theoretical literature on diffusion of innovations helped explore emerging findings and interactions across the *organisational context* and *wider system* domains of the NASSS framework. In particular, it helped analyse factors that influenced local capacity to implement and embed the use home sensors within routine care practice, and how these related to wider regional and national policy contexts.

*The qualitative sampling strategy (purposive and snowball) is appropriate, but further detail on how saturation or thematic sufficiency was assessed would strengthen the methodological transparency. Additionally, the role of gatekeepers in shaping participant recruitment warrants reflection.*

**Response:** As this was a rapid evaluation conducted under tight time constraints, the study was not designed to achieve full data saturation. Instead, the focus was on obtaining a diverse sample of key stakeholders to generate timely insights for policy and practice. We have added clarifying text to the *Methods* section in this regard. Additionally, reflections on the role of organisational gatekeepers is included in the *strengths and limitations* section of the

*Discussion*. *The qualitative analysis is rather scant and opaque (what does [NASSS] 'used adaptively' mean?). Similarly, any triangulation details (between method etc) is lacking.* **Response: **NASSS was used adaptively, in that it was used to guide the development of the case narrative documents by drawing on relevant domains to structure and interpret data within each case, rather than applying each domain as a coding framework. Within our mixed-methods approach, qualitative and quantitative data were mutually informing, and were integrated iteratively to develop the case narrative document. We have added text to the *Methods* section to clarify this aspect of the analysis.

*The economic analysis is presented with appropriate caution. But Propensity Score Matching (PSM) methodology requires further clarification:*

*• which covariates were used in the matching process.*

*• how was balance assessed post-matching.*

*• were any sensitivity analyses conducted? Given the considerable uncertainty implied in the findings, this would seem appropriate.*

**Response:** We have added text to expand this section, providing details on the variables included in the PSM model.  We ran a pstest in Stata 19SE which demonstrated that there were no significant differences in the treatment and control groups across the characteristics that we included in the PSM model. This suggests that balance was achieved across the treatment and control groups. With regard to the sensitivity analysis, we ran a Rosenbaum Bounds test and for all gamma from 1-10, we found no evidence of endogeneity or bias. This means that our results are robust to changing the levels of gamma, and we can be confident that we have not significantly over or under-estimated our coefficient of interest.

*The manuscript touches on digital exclusion and compatibility with service users’ lives, but a more detailed discussion of equity – especially with regard to under-served populations – would add value. Consider elaborating on how inclusion was addressed in the evaluation design and the implications for future deployments of the TEC.*

**Response:** We agree that this is an important issue warranting further reflection and exploration. We plan to write a dedicated paper on this topic, alongside related studies with the DECIDE programme. In the meantime, we have expanded on this topic in the *Discussion* section, focusing specifically on the multiple and intersecting factors influencing inequalities and the challenges these pose for policy and practice.

*The challenges around data sharing and governance are well-documented. And there were obviously problems in accessing data for analysis. Giving space to a more critical reflection on the systemic implications of these barriers – particularly in relation to policy and LA infrastructure – would increase the manuscript’s contribution and doesn't depend on data (ironically).*

**Response: **This is another topic we plan to explore further in a future paper. However, we have expanded on it in the *Discussion *section to highlight some of the systemic factors and implications related to the practical challenges to conducting an economic evaluation. This discussion also helps to frame related points in the subsequent *recommendations *section. In addition, in response to the minor comments we have updated the article to simplify the lay summary and have replaced abbreviations in Tables 3 & 4 with full statistical terms.
